# Lignin-*graft*-Polyoxazoline Conjugated Triazole a Novel Anti-Infective Ointment to Control Persistent Inflammation

**DOI:** 10.1038/srep46412

**Published:** 2017-04-12

**Authors:** Denial Mahata, Malabendu Jana, Arundhuti Jana, Abhishek Mukherjee, Nibendu Mondal, Tilak Saha, Subhajit Sen, Golok B. Nando, Chinmay K. Mukhopadhyay, Ranadhir Chakraborty, Santi M. Mandal

**Affiliations:** 1 Central Research Facility, Rubber Technology Centre, Indian Institute of Technology Kharagpur, Kharagpur 721302, WB, India; 2 Department of Neurological Sciences, Rush University Medical Centre, Chicago, IL, USA; 3 Special Centre for Molecular Medicine, Jawaharlal Nehru University, New Delhi 110 067, India; 4 OMICS Laboratory, Department of Biotechnology, University of North Bengal, Siliguri-734 013, WB, India

## Abstract

Lignin, one of the most abundant renewable feedstock, is used to develop a biocompatible hydrogel as anti-infective ointment. A hydrophilic polyoxazoline chain is grafted through ring opening polymerization, possess homogeneous spherical nanoparticles of 10–15 nm. The copolymer was covalently modified with triazole moiety to fortify the antimicrobial and antibiofilm activities. The hydrogel was capable of down regulating the expression level of IL-1β in LPS induced macrophage cells, and to cause significant reduction of iNOS production. It supported cellular anti-inflammatory activity which was confirmed with luciferase assay, western blot, and NF-κB analysis. This novel lignin-based hydrogel tested *in-vivo* has shown the abilities to prevent infection of burn wound, aid healing, and an anti-inflammatory dressing material. The hydrogel reported here provides a new material platform to introduce a cost-effective and efficient ointment option after undertaking further work to look at its use in the area of clinical practice.

A wide range of polymeric gel including polyurethane, silicone, polyacrylate, and thermoplastic elastomers are used to suite specialized functions for pharmaceuticals to adhesive coating^1^,^2^. Non-toxic polymers, hydrogel in particular, have a forever requirement in ointment formulation. Ointments are generally oily in nature and intended for versatile applications for protective, therapeutic and prophylactic purposes^3^,^4^. There are several disadvantages in ointments application based on the polymeric matrix used in and improper uses. Adverse side effects like allergies, dryness, lesions and thinning of skin is very common problem in ointment application^5^,^6^,^7^. Moreover, the greasy or high lipidic content in ointment is difficult to remove from skin and also lead to clothe staining occasionally^8^. Highly viscous ointments are distributed over infected area improperly and major concern in drug release which depends upon the solubility of matrix. There are different classes of ointments such as oleaginous base, absorption bases, emulsion bases and water-soluble bases^9^,^10^,^11^. Among them, hydrogels are the best one in term of their compatibility in body fluids. They do not flow like a solid but also allow small molecules to diffuse through it like that of a liquid. Hence, the hydrophilic polymer must have the capacity to hold water molecules. The nature of interconnection between polymer chains is either physical held by electrostatic forces, hydrogen bonds, hydrophobic interactions or chemical by means of permanent covalent bonds.

In the present study, lignin was used as the starting polymeric material in formulating a novel hydrogel having suitable properties to treat external wound with microbial infections. The lignin-based hydrogel was designed to render strong anti-inflammatory activities due to addition of antioxidant and antimicrobial properties^12^,^13^. Lignin is the most abundant renewable bio-macromolecules after cellulose. It is the major component of wood (15–30%), act as a binder between tissues, fibres and pith. It makes randomly cross-linked network arising from enzymatic dehydrogenative polymerization of hydroxylated and methoxylated phenylpropane units^14^,^15^. Lignosulfonate is a by-product of the sulfite pulping process in pulp and paper industry. The anionic sulfonate group increases the hydrophilicity of lignin, much like the phenoxide groups under basic conditions, creating a surface-active species for aqueous media^16^,^17^. Several covalently modification of lignin has been studied by graft polymerization to improve the surface homogeneity and its compatibility with other polymer^18^,^19^,^20^. Although the hydrophilic polymer such as polyethylene glycol can be widely used, but it has limitation for formulation of hydrogels based ointment due to dermal irritation with chronic toxicity in inflamed skin^21^. Therefore, we have selected oxazoline monomer for formation of hydrophilic polyoxazoline graft copolymer onto lignin. The lignin copolymer was tailored to give a wide range of properties including carrier of drug, antioxidant, anti-inflammatory and anti-biofilm activity. Finally, a versatile material of quadrant roles has been developed, capable of providing optimum conditions for the debridement of devitalized tissue to promote wound healing.

## Results

### Characterization of lignin-OTs macroinitiator

The tosylation of lignin was carried out by base catalyzed reaction (Fig. S1a) and characterized with FTIR and ^1^HNMR analysis. The FTIR spectrum (Fig. S1b) of lignin shows absorbance peaks at 1718 cm^−1^which may be attributed to C = O stretching vibration of α-β unsaturated ester linkage in ferulic acid and p-coumeric acid fragment of lignin^22^. Two characteristic peaks are observed at1272 and 1040 cm^−1^ corresponding to the C-O stretching of ether linkage and C-O deformation of O-CH_3_ group, respectively^23^. Additionally, O-H bending was observed with broad absorption at 1203 cm^−1^. Appearance of two new peaks at 1363 cm^−1^ corresponding to S = O stretching and at 1080 cm^−1^ for C-OTs stretching suggests the incorporation of –OTS group in tosyl modified lignin. Moreover, shifting of aliphatic O-H bending peak from 1272 to at 1177 cm^−1^ is a clear evidence of tosylation.

As lignin is a branched polymer with aromatic and aliphatic hydroxyl groups, broad signals from 3 to 4.5 ppm for aliphatic protons and 6.3 to 7.3 ppm for aromatic protons in ^1^HNMR spectrum are observed (Fig. S1c). The appearance of two new doublet strong signals at 7.2 and 7.6 ppm for aromatic proton indicates the incorporation of tosyl group at the aliphatic end OH group. The low intense doublet signal at higher chemical shift at 7.8 and 7.4 ppm indicate the incorporation of tosyl groups present at aromatic end terminal hydroxyl group of lignin. Moreover, shifting of tertiary proton signal from 2.1 to 2.3 ppm indicates the formation of tosyl groups in hydroxyl backbone of lignin. Therefore, it is evident that tosylation reaction in lignin takes place in both aromatic as well as aliphatic hydroxyl functional groups^24^. The degree of tosylation of the lignin macroinitiators, amount of excess tosyl chloride was optimized with increasing feeding value in reaction. The absent of hydroxyl peak (O-H) stretching frequency at 3345 cm^−1^ of lignin in FTIR spectrum (Fig. S2A) indicates full substitution of tosyl group in lignin backbone. Therefore, degree of tosyl substitution in lignin-OTs was obtained approximately 70% from the peak integral ratio of partially substituted lignin macroinitiator against fully substituted macroinitiator in aromatic protons signal at 7.6 ppm in ^1^HNMR spectrum (Fig. S2B).

### Characterization of lignin-*g*-POZ (lig-*g*-POZ) copolymer

**Figure 1:**
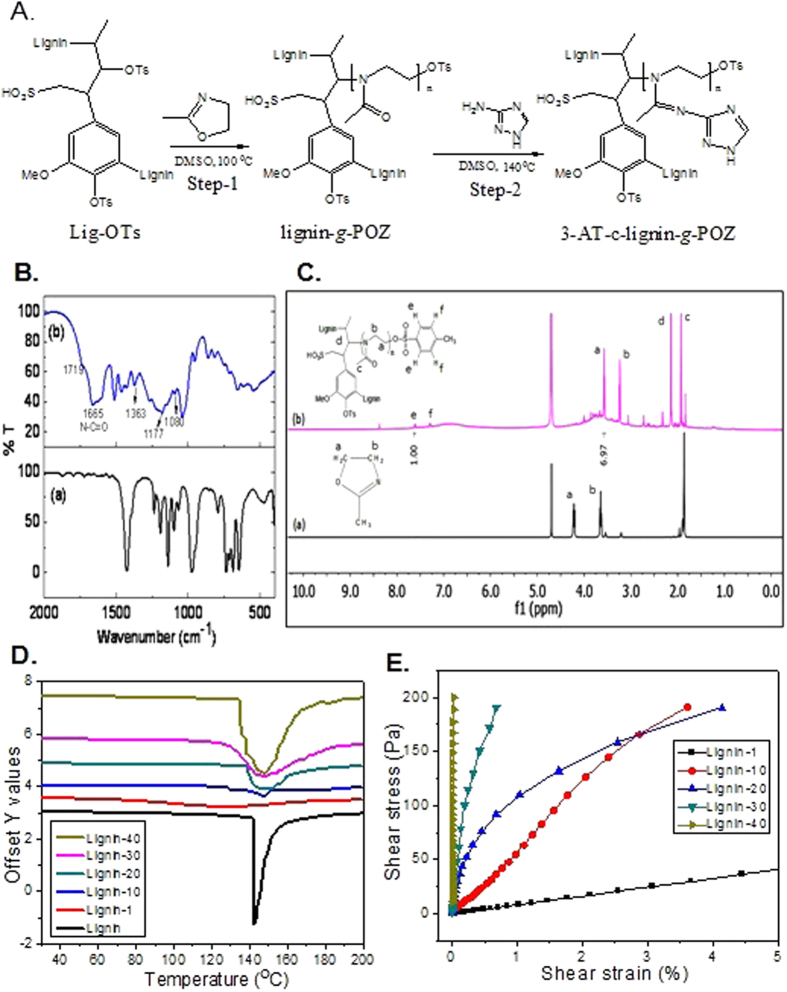
Synthesis and characterization of Lig-*g*-POZ copolymer. Scheme for Lig-*g*-POZ copolymer synthesis from tosylsated lignin (**A**). Characterization by FTIR (**B**) and ^1^H-NMR (**C**) spectrum of 2-methyl oxazoline (a), Lignin-*g*-POZ copolymer (b) in D_2_O. Differential scanning calorimetric analysis of lignin-*g*-POZ copolymer with different weight (%) lignin content in copolymer (**D**). Rheological characterization of lig-*g*-POZ copolymer hydrogel (**E**). Stress vs strain plots of different wt% lignin in copolymer hydrogel.

The aliphatic tosyl groups are more active for the formation of stable aliphatic carbocation to create ring opening polymerization. Therefore, aliphatic tosyl groups act as reactive sites for the formation of cation to initiate oxazoline ring opening. This selective chain growth polymerization of 2-methyl oxazoline is carried out with different weights (1–40%) of tosylated lignin macroinitiator to generate lig-*g*-POZ copolymer (Fig. 1A). The FTIR spectrum (Fig. 1B) of isolated copolymer shows a new peak appearing at 1665 cm^−1^ for C = O stretching frequency which implies the formation of tertiary amide groups by oxazoline ring opening, leads to polyoxazoline chain formation in the copolymer.

The synthesized lig-*g*-POZ copolymer is further characterized by ^1^HNMR analysis (Fig. 1C). The N-methyl oxazoline monomer gives two triplet signals at 4.2 and 3.6 ppm for two ring methylene groups. However, the appearance of two triplet peaks at 3.6 and 3.2 ppm in lig-*g*-POZ copolymer are assigned to the methylene protons of the oxazoline repeating units where signals at 4.2 ppm shifted to 3.2 ppm due to the cleavage of O-CH_2_ bond during the ring opening polymerization. Two doublet signals at 7.2 and 7.6 ppm indicate the presence of -OTs end groups. With varying the lignin macro initiator content from 1 to 40 wt % in the copolymer, the chain length of each grafted polyoxazoline is determined from number-average molecular weight (M_n_) by integration of end group analysis. The resultant polyoxazoline chain length is decreased from 5644 to 282 D in copolymer with increase of lig-OTs macroinitiator from 1 to 40 wt%, respectively (Table S1). The successively decreasing chain length indicates the effective participation of -OTs groups for chain extension during polymerization. Intensity of polyoxazoline methylene proton signals is increased with increasing the chain length (from 40 to 1 wt%) which suggests the efficient modification of more homogeneity in lignin macromolecules (Fig. S3). Therefore, lig-*g*-POZ copolymers is found to be more soluble in common polar solvents such as N-N-dimethyl formamide and dioxane in contrast to unmodified lignin, with a trend of polyoxazoline chain length increased.

### Thermal analysis of lig-*g*-POZ copolymer

The thermal studies by TGA of lignin and lig-*g*-POZ copolymers are shown in Fig. S4. Lignin undergoes three steps degradation in between 200 to 400 °C where the sharp maxima of degradation temperature (T_max_) are observed at 264 °C. A prominent tailing affect is also found beyond 350 °C due to slow degradation of condensed rigid phenolic moieties and strong hydrogen bonding interaction which makes a large proportion (more than 50%) of char. The degradation temperature is drastically changed with the polyoxazoline chain length from 5644 to 282 D in the copolymer i.e. 1 to 40 wt% macroinitiator in lig-*g*-POZ copolymers. The higher polyoxazoline chain length (Mn = 5644 D) in 1 wt% lignin content gives maximum thermal stability than other copolymers in between 200 to 400 °C with a prominent single stage degradation and corresponding T_max_ value is found to be 295 °C. The thermal stability reduces and Tmax shifted at lower temperature from 261 to 212 °C with decrease of polyoxazoline chain length in the copolymers, where lignin weight fraction increases from 10 to 30% successively. However, the higher amount of lignin content i.e, 40 wt%, T_max_ value shifts to higher temperature from 231 °C due to degradation was restricted by breakdown of lignin macromolecules in copolymers. Moreover, the increase of degradation stages from 1 wt% to 40 wt% indicates the loss of homogeneity with decrease of polyoxazoline chain length in copolymer. This can be attributed to the increase of heterogeneous random copolymer structure of lignin in lig-*g*-POZ copolymer.

The DSC analysis of lignin and lig-*g*-POZ copolymer shows a prominent melting peak at 142 °C (Fig. 1D). The lignin does not exhibit a secondary glass transition temperature due to its condensed rigid phenolic moieties and strong intermolecular hydrogen bonding interactions, which restrict the thermal mobility. The copolymer of lignin (1 wt%) with POZ exhibit no prominent melting peak but a secondary glass transition temperature peak observed at 102 °C due to the higher proportion of polyoxazoline chain length (Mn = 5644 D) which increased the thermal mobility. At 10 wt% lignin containing copolymer exhibit a glass transition temperature at 129 °C and corresponding melting temperature is shifted to 147 °C. However, the copolymers from 20 to 40 wt% lignin content has no prominent glass transition temperature, because of the shorter chain length (bellow Mn = 694 D) is not sufficient to enhance the segmental motion in copolymer with temperature. However, the melting temperature also remains unchanged at 148 °C with peak broadening and correspondingly increases of enthalpy. This is might be due to the low chain length of hydrophilic polyoxazoline in lignin surface formed various complex structure by noncovalent interaction during fusion of copolymer at its melting temperature.

### Rheological analysis of lig-*g*-POZ copolymer hydrogel

The stress sweep experiments of lig-*g*-POZ copolymer with different weight percentages of lignin content in hydrogel were performed at a constant frequency 1 Hz (Fig. 1E). The stress-strain plots demonstrate the gelation ability enhances with increasing the lignin content from 1 to 40 wt% in copolymer. This can be explained as efficiency of hydrogen bonding interaction of lignin crosslinker and polyoxazoline chain length. At 1 wt% lignin content (Mn = 5644 D) reveals the weak modulus with high elongation because of low lignin content and high molecular chain length. The modulus of copolymer hydrogel increases from 1 to 40 wt% due to high lignin content and enhances the gelation ability with increase of physical crosslink network. However, the elongation reduces with decrease of polyoxazoline chain length in copolymer from 1 to 40 wt% lignin content copolymer. The result indicates that 10 and 20 wt% lignin in copolymer hydrogel has sufficient elongation but higher gelation ability observed at 20 wt% in comparison to 10 wt% with increasing stress. Moreover, modulus vs strain and modulus vs time plot of 20 wt% hydrogel gives crossover point between elastic modulus and viscous modulus at the minimum strain 0.46% with minimum time required 115 sec for gelation breakdown (Fig. S5). The 30 and 40 wt% of lignin produces strong crosslink structure by intermolecular interaction which shows gelation ability and high modulus value with weak elongation. Therefore, 20 wt% lignin concentrations have been used as optimization of lignin concentration in copolymer which has reasonable gelation ability with efficient visco-elastic behaviour.

### Morphological analysis of lig-*g*-POZ copolymer

**Figure 2:**
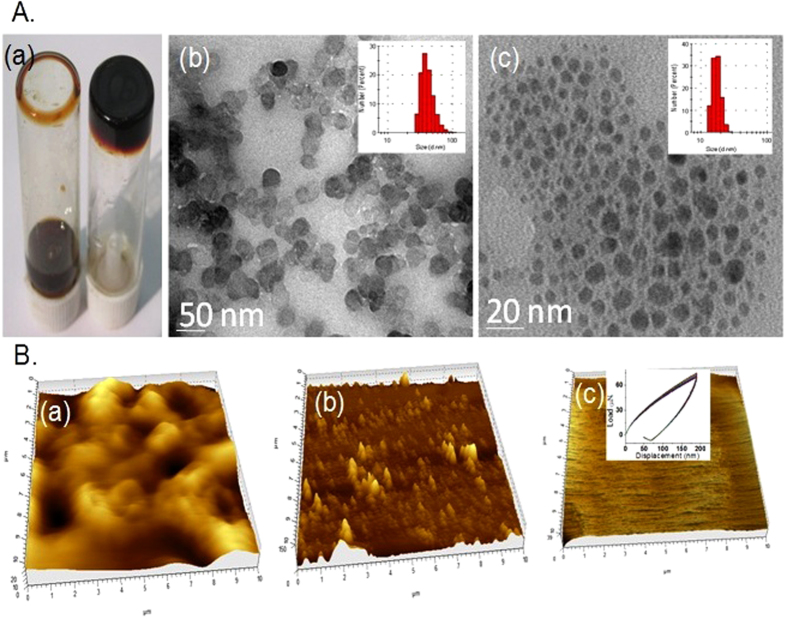
Different images are captured from hydrogel. Visual images of lignin with water (left) and copolymer, lig-*g*-POZ with water, hydrogel (right) (**A**,a). HR-TEM images of aqueous lignin (**A**,b) and lig-*g*-POZ copolymer (**A**,c). Corresponding DLS histogram are given in inset of each TEM image. Atomic Force Microscopic view of lignin (**B**,a), lig-*g*-POZ copolymer (**B**,b). AFM images of lig-*g*-POZ copolymer spin coated thin film (**B**,c) and nanoindentation measurement for hardness is given in inset.

The morphological analysis of lig-*g*-POZ copolymer was characterized by HRTEM and AFM analysis (Fig. 2). The sulfonated lignin gives heterogeneous aggregated spherical nanoparticles due to presence of crosslink aromatic structure (Fig. 2Ab and Ba). However, after surface modification of lignin by 20 wt% polyoxazoline chain, the spherical copolymer nanoparticles tune to outstanding smooth and homogeneous morphology (Fig. 2Ac and Bb) with lignin as antioxidant core^25^. The DLS results also give the average particles size distribution of sulfonated lignin from 50–100 nm which is reduced to 15 to 30 nm after formation of polyoxazoline grafted onto lignin (inset). This result indicates that breakdown of heterogeneous crosslinked network in lignin during surface modification by polyoxazoline chain. Moreover, the surface morphology of 20 wt% polyoxazoline content spin coated copolymer film also strongly suggests the formation of smooth surface on film (Fig. 2Bc). The average hardness 0.0671 GPa with modulus 0.92 GPa of copolymer film from nano-indentation experiment indicates the stability of film under mechanical load (Fig. 2Bc, inset).

### Drug loading and release

The absorbance spectrum of pure AmpB shows high intense band at 328 nm due to its conformational changes in aqueous solvent by different molecular interaction in their self-aggregation structure (Fig. S6a)^26^. The shifting of absorption peak at lower wavelength is increased with their higher order of self-aggregation by complex formation with other molecules. The absorption spectrum of AmpB loaded lig-*g*-POZ copolymer shows an hypsochromic shift from 328 to 325 nm which can be attributed the higher order association of AmpB in aqueous solution that become entrapped inside of lig-*g*-POZ copolymer hydrogel^27^. The drug loading efficiency and corresponding drug loading content in copolymer is obtained to be 84.2% and 16.9%, respectively (Fig. S6b). The drug loading efficiency and corresponding drug loading content in copolymer is obtained to be 84.2% and 16.9%, respectively (Fig. S6b). The release of AmpB gradually increases with increasing the more basic or acidic condition in buffer and found to be 32.5, 21.0, 40.0 and 50.0% after 7 days at pH 8.5, 7.5, 6.5 and 5.5, respectively. This can be explained by the increase of higher diffusion of AmpB in copolymer hydrogel due to their more stable monomeric form rather than oligomer at more basic as well as high acidic medium due to the zwitterionic chemical structure may also broken down with loss of reversibility in both basic and acidic medium.

### Characterization of 3-AT-c-lig-*g*-POZ copolymer

Triazole conjugated lig-*g*-POZ copolymer is synthesized by condensation reaction between 20 wt% lignin containing copolymer and 3-amino-1H-1,2,4 triazole (3-AT), shown in Fig. 1A. The FTIR spectrum (Fig. S7a) of 3-AT gives two characteristics strong band at 1638 cm^−1^ and 1590 cm^−1^ for triazole ring C = N and C = C stretching frequency, respectively. Appearance of new peak at 1695 cm^−1^ for aliphatic C = N stretching and disappearance of C = O stretching frequency at 1656 cm^−1^ of oxazoline chain indicates the formation of aliphatic imine linkage with primary amine in lig-*g*-POZ copolymer. The ^1^HNMR spectrum (Fig. S7b) show the appearance of a new signal at 7.76 ppm for triazole ring methine proton suggests their successful attachment to lig-*g*-POZ copolymer chain. The percent of triazole grafting (96%) is also determined by the integration of methylene proton (3.52 ppm) in oxazoline unit against triazole ring methine proton signal at 7.76 ppm.

### Antioxidant activity

Antioxidant activities of lignin, lig-*g*-POZ and 3-AT-c-lig-*g*-POZ copolymers are determined by DPPH radical assay^28^,^29^. The Torolox, a strong antioxidant was considered as control for comparative measure of efficacy of lignin copolymers^30^. The free radical scavenging activity (Fig. S8a) of lignin, lig-*g*-POZ and 3-AT-c-lig-*g*-POZ is found to be 10.5, 7.6, 7.0% respectively. The free radical scavenging ability of lignin extensively depends on phenolic hydroxyl groups only while the role of aliphatic -OH remains unaffected. Therefore, the total phenolic hydroxyl groups in copolymers were quantified by Folin-Ciocalteu test (Fig. S8b). Presence of free phenol groups in lignin, lig-*g*-POZ and 3-AT-c-lig-*g*-POZ copolymer is also found to be 9.6, 4.6 and 4.2 of gallic acid equivalent gm.100 g^−1^. The decrease of phenolic hydroxyl groups indicates the free radical scavenging activity much lower in lig-*g*-POZ hydrogel and 3-AT-c-lig-*g*-POZ copolymer.

### Lignin, lig-*g*-POZ and 3-AT-c-lig-*g*-POZ inhibited the LPS induced NO production in mouse RAW cells

LPS is a potent systematic inducers^31^. Several reports indicated that LPS are a potent inducer of NO production in macrophages^32^. Specifically, the inducible nitric oxide synthase is upregulated during inflammation and infection, and up-regulation can be sustained over a prolonged period culminating in the production of large quantities of NO. To determine the anti-inflammatory effect of lignin, lig-*g*-POZ and 3-AT-c-lig-*g*-POZ, different concentrations of lignin derivatives on LPS-induced NO production were investigated in RAW 264.7 macrophages. As shown in Fig. S9A, all three compounds dose dependently markedly inhibited the NO production in RAW cells. Lig-*g*-POZ is more potently inhibited the NO production compare to 3-AT-c-lig-g-POZ in RAW cells (Fig. S9A). Lignin, lig-g-POZ and 3-AT-c-lig-g-POZ up to 50 μg/mL did not affect on cell viability determined by MTT metabolism and LDH release (Fig. S9B and C). Further MTT measurement was also performed using final formulated nanocomposite against HEK293 cell line and MTT conversion activity was slightly reduced (<25%) at concentration of 60 μg/mL (S9 D).

### Lignin derivatives inhibit LPS-induced expression of iNOS and proinflammatory cytokine IL-1β, TNF-α and IL-6 in mouse macrophages and RAW cells

**Figure 3:**
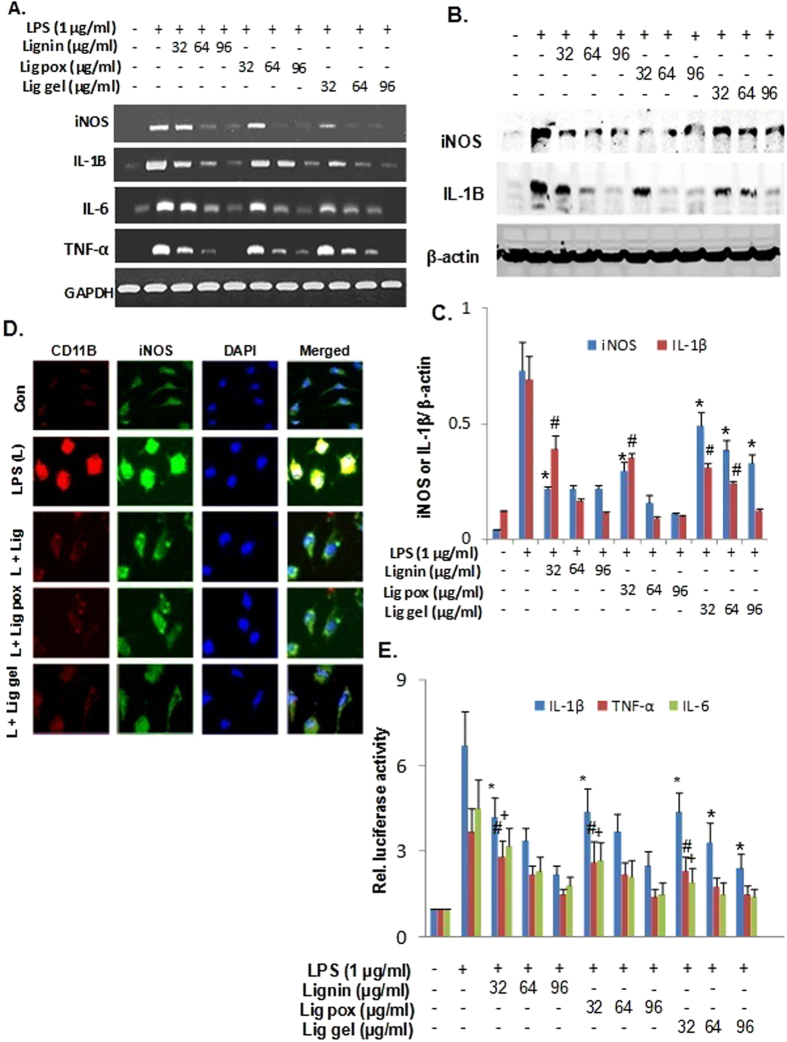
Effect of lignin derivatives on the expression of iNOS, TNF-α, IL-6 and IL-1β in Raw cells. The abbreviations ‘Lig pox’ and ‘Lig gel’ represents the treatment with compound ‘lig-*g*-POZ’ and ‘3AT-c-lig-*g*-POZ’ respectively. Raw cells were incubated with different concentrations of lignin, lig-*g*-POZ and 3-AT-c-lig-*g*-POZ under serum free conditions. Then, immediately after 2 h of incubation, the treated medium from culture plate was wiped out and gently washed with PBS(1X) for twice before the stimulation with 1 μg/ml of LPS and after 6 h of treatment, total RNA was analyzed for the expression of iNOS, IL-1β, TNF-α, IL-6 and GAPDH by semiquantitative RT-PCR and quantitative real time- PCR (**A**). Raw cells were preincubated with different concentrations of lignin, lig-*g*-POZ and 3-AT-c-lig-*g*-POZ for 2 h under serum free conditions were stimulated with 1 μg/ml of LPS and after 18 h of treatment, iNOS, IL-1β and total β-actin proteins were analyzed by western blot (**B**). The western blot bands are scanned for quantification and represented as relative expression (**C**). Data are mean ± S.D. of three separate experiments.*p < 0.05 *vs* cells treated with LPS, and ^#^p < 0.05 *vs* cells treated with LPS in the absence of lignin derivatives. Mouse macrophages were preincubated with 32 μg/ml of lignin, lig-*g*-POZ and 3-AT-c-lig-*g*-POZ for 2 h under serum free conditions were stimulated with 1 μg/ml of LPS and after 18 h cells were immunostained with iNOS, and IL-1β (**D**); scale bars represent 20 μM. DAPI was used to visualize nucleus. Twenty-four hours after transfection, cells were preincubated with different concentrations of lignin, lig-*g*-POZ and 3-AT-c-lig-*g*-POZ for 2 h under serum free conditions were stimulated with 1 μg/ml of LPS for 6 h (**E**). Firefly and Renilla luciferase activities were determined by Dual Luciferase Kit (Promega) following the manufacturer’s protocol. Data are mean ± S.D. of three separate experiments. *p < 0.05 *vs* cells treated with LPS, ^#^p < 0.05 *vs* cells treated with LPS, ^+^p < 0.05 *vs* cells treated with LPS.

To determine the effect lignin, lig-*g*-POZ and 3-AT-c-lig-*g*-POZ on the activation of mouse macrophages, RAW cells were exposed to different concentrations of lignin derivatives (32–96 μM) for 2 h, after removal of polymer treated medium, stimulation was done with LPS for 6 h, RNA was harvested for semi-quantitative PCR analysis (Fig. 3A). It was observed that LPS distinctly induced mRNA expression of iNOS and proinflammatory cytokine IL-1β, TNF-α and IL-6 in RAW cells. As evident from the western blot in Fig. 3B and C, lignin, lig-*g*-POZ and 3-AT-c-lig-*g*-POZ dose dependently inhibited the iNOS and IL-1β production in RAW cells. Lignin derivatives significantly inhibited at lower doses also. Similarly, immunofluorescence analysis also shows that lignin, lig-*g*-POZ and 3-AT-c-lig-*g*-POZ markedly suppressed the LPS-induced iNOS and IL-1β protein production in mouse primary macrophages (Fig. 3D). Next, we tested whether lignin, lig-*g*-POZ and 3-AT-c-lig-*g*-POZ transcriptionally regulated IL-1β, TNF-α and IL-6 expression. Cells were transfected with the reporter plasmid PGL3 containing IL-1β, TNF-α and IL-6 promoter fragment. As evident from Fig. 3E, lignin derivatives dose-dependently inhibited IL-1β, TNF-α and IL-6 promoter-driven luciferase activity in RAW cells.

### Effect of lignin derivatives on the activation of proinflammatory transcription factor NF-κB in mouse RAW cells

**Figure 4:**
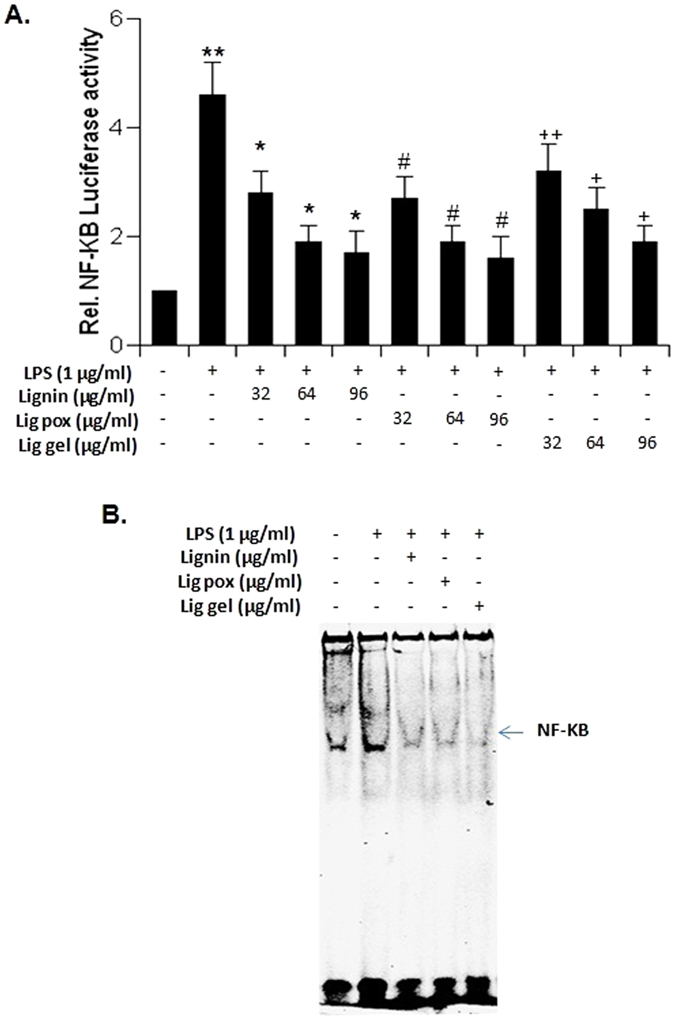
Effect of lignin derivatives on NF-κB-dependent luciferase activity in LPS-stimulated RAW cells. The abbreviations ‘Lig pox’ and ‘Lig gel’ represents the treatment with compound ‘lig-*g*-POZ’ and ‘3AT-c-lig-*g*-POZ’ respectively. RAW cells plated at 50-60% confluence in twelve-well plates were cotransfected with 0.5 μg of pNF-κB-Luc and 25 ng of pRL-TK. After 24 h of transfection, cells were incubated with 60 μg of lignin derivatives for 2 h and then stimulated with LPS for 6 h under serum free condition. Firefly and renila luciferase activities were obtained by analyzing the total cell extract. Results represent three independent experiments and Data are mean ± S.D. of three separate experiments. The level of significance *p < 0.05 and**p < 0.01 *vs* cells treated with LPS, ^#^p < 0.05 and ^##^*p* < 0.01 *vs* cells treated with LPS, ^+^p < 0.05 and ^++^*p* < 0.01 *vs* cells treated with LPS(A). Cells preincubated with 60 μg of lignin derivatives for 2 h were stimulated with LPS under serum-free condition. After 1 h of stimulation nuclear extracts were prepared and subjected to EMSA for the detection of NF-κB as described in Materials and Methods (**B**).

To understand the basis of suppression of proinflammatory molecules by lignin, lig-*g*-POZ and 3-AT-c-lig-*g*-POZ, examined whether the activation of NF-κB in LPS- stimulated RAW cells was also regulated by lignin derivatives. Activity of NF-κB was monitored by DNA binding activity of NF-κB^33^,^34^. DNA binding activity of NF-κB was evaluated by the formation of distinct and specific complex in a gel shift DNA binding assay. Activation of NF-κB was monitored by transcriptional activity using the expression of luciferase reporter construct. This inhibitory effect was dose-dependent, and the maximum inhibition was found at 60–100 μg/mL concentration of lignin derivatives (Fig. 4A). Treatment of RAW cells with LPS resulted in the induction of DNA binding activity of NF- κB, whereas lignin derivatives markedly suppressed the DNA binding activity (Fig. 4B).

### Antimicrobial activity

**Figure 5:**
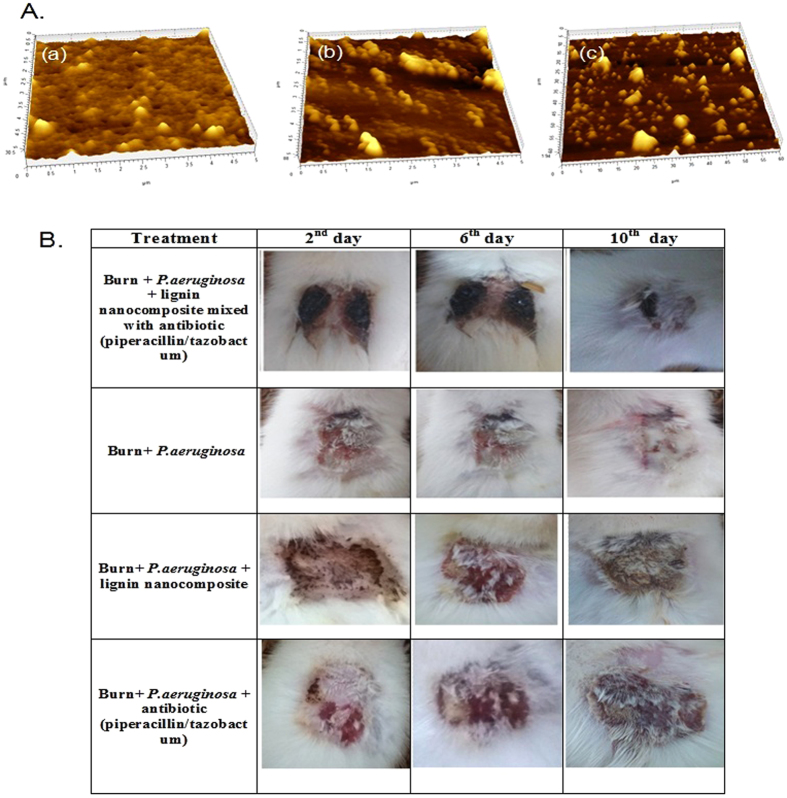
Biofilm and Progress of infected (*Pseudomonas aeruginosa* HW01) burn-wound healing. Biofilm formation by the pathogen, *Pseudomonas aeruginosa* HW01was allowed for 48 h in polysterene plate and after washing the medium, new medium was introduced with 3-AT-c-lig-*g*-POZ (64 μg/mL) (**A**,b) and 3AT-c-lig-*g*-POZ with antibiotics (piperacillin/tazobactum) (**A**,c) and control without any chemical agents (**A**,a). After overnight (16 h) treatment, the biofilms were visualized in AFM microscope and captured the images. *In-vivo* experiment on wound healing activity by the formulated anti-infective ointment (**B**) [lignin nanocomposite mixed with antibiotic (piperacillin/tazobactum); (1 mL mixture contained 64 μg lignin nanocomposite and 56/7 μg piperacillin/tazobactam)] (1^st^ row) compared with (i) control (2^nd^ row); (ii) treated with lignin nanocomposite (64 μg/mL) alone (3^rd^ row); iii) treated with antibiotic (piperacillin/tazobactum) (1 mL contained 56/7 μg piperacillin/tazobactam) (4^th^ row).

All the compounds, lignin, lig-*g*-POZ and 3-AT-c-lig-*g*-POZ were tested for antimicrobial activity^35^ against two Gram-positive bacteria, S*. aureus, S. epidermidis*, four Gram-negative bacteria as *E. coli, P. aeruginosa, S. typhi, K. pneumonia* and two fungal strains *C. albicans, C. tropicalis* (Table S2). The concentrations ranged from 0.97 μg/ml to 1 mg/ml. No significant growth reductions were observed in tested concentration range for lignin and lig-*g*-POZ copolymer, whereas growth was significantly inhibited with the treatment of 3-AT-c-lig-*g*-POZ copolymer. AFM analysis (Fig. 5A) of untreated and 3-AT-c-lig-*g-*POZ treated *P. aeruginosa* biofilm has revealed significant difference in thickness of the biofilm structure from 30 nm (untreated) to 8 nm (treated) after 12 h of incubation. Thus, treatment with 3-AT-c-lig-*g*-POZ could prevent the growth of biofilm by allowing drugs to act on the cells. The attachment of bacterial cells in polystyrene plate was quantified using crystal-violet stain (Fig. S10).

### *In-vivo* wound healing

An incomplete-thickness burn has resulted in the rats from flame burning process adapted in this study. Residual wound area (where wound area on the day of infection taken as 100%) measured at every 2 days interval, have been shown to progressively decrease to 0.69% ± 0.04 at the end of 14 days in case of Gr-II animals where burn wound infected with *P. aeruginosa* was treated with lignin nanocomposite mixed with dry piperacillin/tazobactum as anti-infective ointment (Fig. 5B). The residual wound area (infected with *P. aeruginosa*) of the untreated animals (Gr-V treated with a spray of PSS alone) have shown to decrease to only 72.92% ± 2.70 (Fig. S11). Higher re-epithelialization and re-appearance of fur in the burn-wound area was also evident in case of animals treated with anti-infective ointment containing piperacillin and tazobactum (Fig. S12). The PRWA data for animals comprising both control (Gr-V) and test animals (Gr-I, II, III & IV) on statistical analyses have revealed the following: (i) the difference in treatment pair, Gr-V (control) vs Gr-I (treated with lignin nanocomposite alone) was insignificant (Bonferroni p-value = 0.136; Scheffe p value = 0.313); (ii) Gr-V (control) vs Gr-II (treated with lignin nanocomposite mixed with dry piperacillin/tazobactum) was significantly different in both Bonferroni and Holm simultaneous compared results (p value = 0.0000e + 00; Bonferroni/Holm inference, **p < 0.01; Scheffe p value = 1.1102e-16, Scheffe inference **p < 0.01); (iii) in treatment pairs Gr-II vs Gr I, Gr-II vs Gr-III (treated with piperacillin/tazobactum alone), and Gr-II vs Gr-IV (treated with silver nitrate and chlorohexidine gluconate cream) were significantly different (p-value in both Bonferroni and Holm tests for three different comparisons was 0.0000e + 00; inference **p < 0.01; Scheffe p value and inference for all three test pairs were 1.1102e-16 and**p < 0.01 respectively). The pathogen-load for the particular strain of *P. aeruginosa* HW01 (ascertained qualitatively) progressively reduced to nil in the 4th day following burn, while detection of viable bacteria were still observed in the burn wounds of the untreated animals (Table S3A). We have observed that burn injury with simultaneous infection of *P. aeruginosa* resulted log- fold increase from the normal level of serum CRP (normal level = ˜0.6 mg/ml) on day 2 after burn injury in both treated and untreated animals. The increased CRP level was found to subside to normal level with resolution of inflammation in the animals treated with anti-infective ointment. In control animals, the sign of infection was still persistent on the 4th day post-burn infection (Table S3B).

## Discussion

**Figure 6:**
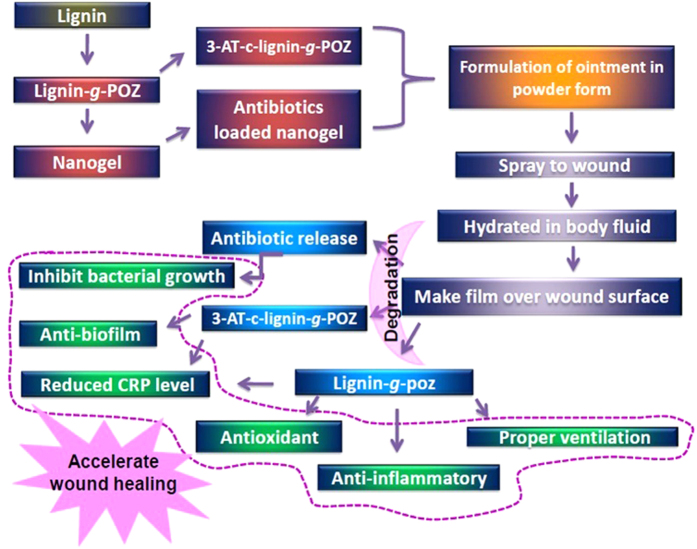
Lignin graft polyoxazoline copolymer conjugated triazole based nanogel preparation and their versatile applications to accelerate wound healing process.

The applications of renewable polymers based hydrogels in biomedicine have added novelty to sustainable alternatives in health care. Here, we have developed a physically crosslinked, biocomapatible hydrogel from renewable lignin after surface modification with a hydrophilic polyoxazoline chain by chemical grafting approach which showed versatile benefits to speed up the wound healing process (Fig. 6). Lignin is a highly branched polyphenolic core structure with various hydrophobic (aromatic, aliphatic) moieties. The physico-mechanical properties of hydrogels were drastically altered with variation of hydrophilic segmental chain length. The hydrogen bonded crosslinked network massively built with 10 to 20 wt% lignin content copolymer indicates the better performance with perfect elasticity. The morphological structures of hydrogel gives highly ordered, homogeneous spheres of nanostructure with diameter 15–30 nm indicates the formation of nanogel which served as the carrier of an antimicrobial drugs. Here, AmpB was used to evaluate the loading and sustain release from copolymer. AmpB is a pH sensitive zwitterionic amphiphilic molecule which contains hydrophobic conjugated polyene chain with several hydrophilic hydroxyl, carbonyl, and amide groups. Therefore, interaction is possible through hydrogen bonding, hydrophobic stacking interaction with lig-*g*-POZ copolymer. The synthesized lig-*g*-poz copolymer did not exhibit antimicrobial activity. Therefore, to enhance the better therapeutic efficacy of copolymer, triazole moieties were conjugated on its oxazoline backbone. The newly synthesized 3-AT-c-lig-*g*-Poz showed significant antimicrobial activity. It has shown to be most effective against the fungal strains which could be due to the graft of triazole in polymer chain, as triazole generally inhibits the action of specific enzyme C-14 demethylase^36^ which impart for sterol production in fungal cells. It is also observed that treatment of triazole copolymer resulted in drastic reduction in the growth of biofilm. Therefore, we realized that 3AT-c-lig-*g-*POZ is also necessary with lig-*g*-POZ copolymer for a better wound healing activity in ointment formulation.

The copolymer lig-*g*-POZ showed relatively higher antioxidant and anti-inflammatory activity in comparison to 3AT-c-lig-*g-*POZ. Inflammation is the immune response to pathogens or harmful agents. Body starts to encounter infection, and releases inflammatory molecules. Among several transcription factors, NF-κB is one of the most important transcription factor involved in the transcription of maximum number of proinflammatory molecules. Since lignin, lig-*g*-Poz inhibited LPS-induced activation of mouse TNFα, IL-1β and IL-6 promoter and markedly suppressed the LPS-induced activation of NF-κB. The results demonstrated that lignin derived copolymers, lig-*g*-POZ and 3-AT-c-lig-*g*-POZ are potent anti-inflammatory agents were determined by NF-κB-dependent luciferase assay (Fig. 4A). It may be used as an effective material for the treatment and prevention of inflammation. The CRP level in plasma was also evaluated during the treatment with ointment. The plasma concentration of CRP solely depends on rate of its synthesis, which in turn increases many folds within 48 hours of injury, infection or tissue damage. CRP in addition to WBC count is the most widely used parameter for diagnosis of infection. Like human CRP (hCRP), rat CRP (rCRP) plays an important role in host defence as a proinflammatory mediator and activator of complement pathway^37^. This acute phase protein is produced by the hepatocytes in response to IL2, IL1 or TNFα generated during acute infection or tissue damage. The results of the studies, which are shown in Table S3, Figs 5, and S11 have shown clear indication that the drug impregnated anti-infective ointment rendered better protection in comparison to the treatment with only antibiotics or nanocomposite alone. The p-value corresponding to the F-statistic of one-way ANOVA of five independent treatments was lower than 0.05 suggesting that the one or more treatments were significantly different. Hence, the Turkey HST test, Scheffe, Bonferroni and Holm multiple comparison tests were followed. These post-hoc tests have identified which of the pairs of treatments were significantly different from each other. The burn wound healing in animals treated with lignin-based nanocomposite alone or with antibiotic alone in comparison to treatment with lignin nanocomposite mixed with dry piperacillin/tazobactum *in vivo* have yielded statistically significant differences.

The present study was intended to elucidate progress of healing burn-wound (infected with a pathogenic MAR strain of *P. aeruginosa*) with antibiotic impregnated anti-infective topical ointment compared to untreated animals with infected burn-wound flushed with sterile PBS with same antibiotics. It is apparent from this study that the animals suffered minimal thermal injury while proved susceptible to infection with *P. aeruginosa* and enabled us to control burn-wound infection or to evaluate therapeutic efficacy of a novel anti-infective ointment. The surface inoculation of the freshly burned skin with *P. aeruginosa* was found to be significantly influenced by topical therapy. The CRP level was found to be elevated in both treated and control animals up to 2 days post-infection. The fall in elevated CRP level, in treated animals during 4 days post-infection, indicated recovery vis-a-vis efficacy of the topical ointment. The present study has also indicated an additional bio-medical quality of our anti-infective ointment that is very important in case of nursing burn wounds. Repeated dressings and application of fresh ointment over the burnt region is not required with our innovated ointment.

We may conclude that the synthesized lignin-*graft*-polyoxazoline copolymer has the efficiency to form hydrogel. Among different weight contents of lignin tested to produce the copolymer, 20 wt% lignin was found to be the most appropriate content for hydrogel formation having efficient viscoelastic properties. The hydrogel has revealed the formation of spherical copolymer nanoparticles with an average size of 10–15 nm. It has been tested for efficacy as ointment fortified with antimicrobial and anti-biofilm properties (after conjugation with triazole moiety), and significant drug delivery efficiency. In clinical settings, inflammations are thought to be the major hurdle in wound healing. The *in vitro* study using mouse macrophage cells treated with LPS to induce inflammation revealed that lignin coplymer has the ability to reduce the inflammation by down regulating the gene expression level of iNOS and IL-1β. The hydrogel showed free radical scavenging activity which provided benefit to infected tissue in healing process because reactive oxygen species are also an impede in recovery. Therefore, the multifunctional benefits of this novel lignin based hydrogel may be used in future generation ointment formulation in successful and efficient wound therapy.

## Methods

### Materials

Sulfonated lignin, Tosyl chloride, 2-methyl oxazoline, 3-amino-1H-1,2,4 triazole was purchased from Sigma Aldrich, USA. All solvents were of analytical grade from Merck (India). Fetal bovine serum, Hank’s balanced salt solution (HBSS) and Dulbecco’s modified Eagle’s medium (DMEM)/F-12 were from Mediatech, Valley Park, MO, USA. Dulbecco’s Modified Eagle Media (DMEM-F12), Fetal bovine serum (FBS), Antimycotic antibiotic, L-glutamine, 0.05% trypsin-EDTA solution and Phosphate Buffered Saline (PBS) were purchased from high media, India.

### Synthesis of tosylated lignin (Lignin-OTs) macroinitiator

Tosylated lignin was prepared according to procedure Diop *et al*. in aqueous medium^38^. One gram of lignin was dissolved in 10 mL distilled water and stirred for 1 hour after addition of 0.0025 molar (250 mg) of triethyl amine at 0–5 °C. Then 0.0025 molar (475 mg) of tosyl chloride (TsCl) in THF solution (10 mL) was added drop wise and the reaction was carried out 10 hours. The mixure was concentrated and precipitated with ethanol. The product was washed with deionized water ethanol (1:1) mixture and dried under vacuum oven at 60 °C. Further, tosylation reaction of both the aliphatic and the phenolic hydroxyl groups was performed by dissolving 1 gm of lignin in water and excess triethyl amine (2 mL) was added in it at 0–5 °C. The mixture was cooled in an ice bath and 2 gm of tosyl chloride was slowly added drop wise. The reaction mixture was cooled at room temperature with stirring for 24 h. The lignin-macroinitiator was recovered and purified as described above. Therefore, the degree of tosylation of above reaction (70%) was determined by the peak integration of partially tosylated lignin divided by integration of fully tosylated lignin against aromatic proton at 7.6 ppm.

### Synthesis of lignin-*graft*-polyoxazoline or lig-g-POZ copolymer by ring opening polymerization (Lignin:oxazoline 20:80)

Tosylated lignin (500 mg, 10 wt%) was used as a macroinitiator to carry out ring opening polymerization of 2- ethyl oxazoline monomer (2 gm, 90 wt%) in 10 mL DMSO solvent for preparation of lignin grafted polyoxazoline^39^. The round bottom flask was sealed with rubber stopper, immersed into an oil bath at 100 °C and the reaction carried out under nitrogen atmosphere for 10 hrs with stirring. An aliquot was taken out from the reaction mixture and percent of conversion (81%) determined by integration of the 2-ethyl oxazoline ring methylene proton at 4.22 ppm against integration of polyoxazoline methylene proton signal at 3.53 ppm. The reminder crude reaction mixture was precipitated in isopropyl alcohol and product was separated by centrifugation at 2000 r.p.m. The product was centrifuged three times with same procedure and finally, dried at 120 °C under vacuum oven for 2 days. The average molecular weight of polyoxazoline chain length was determined by end group integration of tosyl aromatic proton at 7.46 ppm against peak integration of oxazoline (CH_2_-N-) repeating unit at 3.6 ppm in copolymer chain (Mn = 593 Da).

### Synthesis of 3-amino-1H-1,2,4 triazole conjugated lig-g-POZ or 3-AT-c-lig-g-POZ copolymer

Lig-*g*-POZ copolymer (100 mg) was dissolved in 5 mL DMF solvent in a reaction tube and 30 mg 3-amino-1H-1,2,4 triazole (3AT) added in the mixture. The solution was refluxed with condenser at 120 °C for 12 hrs with stirring under nitrogen atmosphere. The reaction mixture was precipitated in isopropyl alcohol and product was separated by centrifugation at 2000 r.p.m. The purified product was dried at 150 °C under vacuum oven for 2 days. The percent of triazole grafted (96%) is also obtained by the integration of methylene proton (2 H) at 3.56 ppm in oxazoline unit against triazole ring methane proton (1 H) signal at 7.76 ppm unit.

### Characterization of lig-*g*-POZ and 3-AT-c-lig-*g*-POZ copolymers

For FTIR analysis, The KBr pressed disc technique (2–4 mg of sample and 200 mg of KBr) was used. Data acquired in Shimadzu 8400 FT-IR spectrophotometer. Absorbance spectra were obtained from 4000 to 400 cm-1 with a 4 cm-1 resolution, Background spectra were also collected and subtracted. The ^1^H spectra were taken on a Bruker DPX200 (500 MHz for 13 C and 200 MHz for 1 H) in D_2_O solvent using tetramethylsilane (TMS) as an internal standard. Both cases 5 mg/mL concentrated solutions were prepared. All signals were referenced to TMS within ± 0.1 ppm. The thermo oxidative stability of pure lignin and lig-*g*-POZ with different weight (%) lignin content copolymer were carried out using a TGA Q50 thermal analyzer of TA Instruments, USA. All the experiments were performed by heating the sample at a rate of 10 °C/min, in nitrogen atmosphere, from room temperature to 600 °C. DSC studies of the pure lignin and lig-*g*-POZ copolymer with different weight (%) lignin content were performed using a DSC Q100, of TA instruments make, USA. Approximately 10 mg of the samples was placed in an aluminum crimple and sealed with the help of a hand press prior to placing it on the sample platform of the instrument. The scanning was carried out in the temperature range of 30 °C to 200 °C at a heating rate of 10 °C/min in a stream of continuous supply of dry nitrogen.

### HR-TEM analysis

HR-TEM was performed in JEOL JEM 2100 instrument. One mg/mL concentration of lignin and lig-b-POZ copolymer in aqueous solution was drop cast on a Cu-grid. After drying the grid at ambient temperature, the samples were directly imaged under TEM. To record the AFM images of lignin and Lig-g-POZ, 10 μL of 1 mg/mL concentrated samples in aqueous solution was directly spotted on the glass cover slip as a drop-caste method. Surface morphology was studied by using AFM and scanning electron microscope JEOL JSM 5800) with an accelerated voltage between 5–20 kV. Moreover, the bio-flim images were also carried out in same instruments. The dynamic light scattering experiment was performed with an argon ion laser system (DLS, Malvern Instruments, Series 4700) at 25 °C. One mg/mL concentration of aqueous solution of lignin and lig-g-POZ copolymer was used for analysis at pH 7 in aqueous media. DLS measurements of average particle size were performed with scattering angle of 90°. The standard deviation was calculated from more than twenty DLS measurements over a time period sufficient to reach equilibrium.

### Rheological analysis

Rheological properties of copolymers were carried out by Bohlin Gemini (Malvern, UK) instrument with controlled-stress rheometer using 20 mm diameter parallel plate geometry with a constant tool gap of 100 μm. The gel sample was located on the lower plate, and the stress sweep measurements were performed at a constant frequency 1 Hz in the linear viscoelastic range.

### Nanoindentation

Two mm thickness films of 20 wt% lig-*g*-POZ copolymer film were prepared by drop casting techniques. The surface hardness was measured with a TI 950 TriboIndenter, Hysitron Inc., USA at room temperature with a varied load at constant displacement 200 nm.

### Determination of drug loading and release study

Amphoterecin- B (AmpB) loaded into lig-*g*-POZ copolymer nanogel was prepared by dialysis method^40^. A mixture of copolymer (25 mg) and AmpB (5 mg) were dissolved in 5 mL double distilled water and stirred for 2 hrs. Then, solution was transferred to a 2000 Da molecular weight cutoff dialysis bag and dialyzed for 14 hrs to remove the impurities and free AmpB. The absorbance of AmpB loaded copolymer solution (100 μg/mL) was measured at 328 nm by UV-Visible spectrophotometer. A calibration curve was constructed using different concentrations (1–100 μg/mL) of free drug in buffer. Drug loading content (DLC) and drug loading efficiency (DLE) were calculated from the following equation:


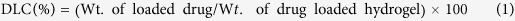



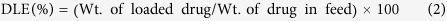


To obtain the *in vitro* drug release profile, 3 mL drug loaded hydrogel (3 mg/mL) were sealed in a dialysis tube (molecule cut-off 3,000) and incubated in 20 mL buffer solutions at 27 °C under stirring at a speed of 80 rpm. At regular time intervals, the 3 mL was removed and replaced by fresh PBS to maintain sink conditions. The drug concentration in solution was calculated following on absorbance at 330 nm for AmpB by UV−visible spectrophotometer. The cumulative drug release percent (E_r_%) was calculated by the following equation:


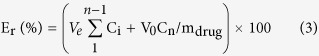


Where m_drugs_ represents the amount of drugs in the hydrogel, V_0_ is the whole volume of the release media (V_0_ = 20 mL), V_e_ is the volume of the replace media (V_e_ = 20 mL), and C_n_ represents the concentration of drugs in the nth sample. Where release media (V_0_ = 100 mL), V_e_ is the volume of the replace media (V_e_ = 3 mL).

### Antioxidant assay

The antioxidant activity of lignin, lig-*g*-POZ and 3-AT-c-lig-*g*-POZ copolymer were estimated using DPPH (2,2-diphenyl-1-picrylhydrazyl) radical scavenging protocol^41^. For a typical reaction, 190 μL of 100 μM DPPH solution (final concentration was 1% in ethanol) was mixed with individual 10 μL sample (2 μg/mL) solution in triplicates. The reaction mixture was incubated in the dark for 15 min and thereafter the optical density was recorded at 515 nm absorbance (A) against the blank. For control, 190 μL of 100 μM DPPH solution in ethanol was mixed with 10 μL of distilled water. The decrease in optical density of DPPH in relation to control was used to calculate the antioxidant activity, as percentage of inhibition in equation 4.


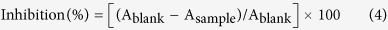


### Total polyphenol content (TPC)

The total polyphenol content was determined by spectrophotometry according to the method described by using gallic acid as standard in water^42^. One mL of all samples (100 μg/mL) was added in to separate tubes containing 1.0 mL of a 1/10 dilution of Folin-Ciocalteu’s reagent with water. Then, 1.0 mL of a potassium carbonate (7.5% w/v) solution was added in mixture and kept at room temperature in dark for 50 mins. Absorbance was monitored at 765 nm. The concentration of all samples were determined from a standard curve of gallic acid ranging from 5 to 100 μg/mL (pearson’s correlation coefficient: r^2^ = 0.9769) and TPC was expressed as gallic acid equivalents (GAE) in g/100 g material.

### Isolation of mouse peritoneal macrophages

Macrophages were isolated sterile RPMI 1640 medium containing 1% FBS and antibiotic-antimycotic mixture (Sigma) from peritoneal lavage from mice as described earlier^43^,^44^,^45^. Cells were washed gently thrice with RPMI 1640 medium at 4 °C. Cells were maintained at 37 °C in a humidified incubator containing 5% CO_2_. Cells were plated in same medium and after 1 h, non-adherent cells were removed by washing.

### Immunostaining

Immunostaining was performed as described earlier^44^,^46^,^47^. In brief, coverslips having 200–300 cells/mm^2^ were fixed with 4% paraformaldehyde for 15 min, treated with cold ethanol (−20 °C) for 5 min and rinses twice with PBS (1X). Samples were blocked with 3% BSA in PBS containing Tween 20 (PBST) for 30 min and incubated in PBST containing 1% BSA with rabbit anti-iNOS and IL-1B (1:200 each). Further cells were gently washed with PBST (15 min each and three times) and incubated with Cy5 and Cy2 (Jackson ImmunoResearch, West Grove, PA, USA). The samples were mounted and observed under a Bio-Rad (Hercules, CA, USA) MRC1024ES confocal laser-scanning microscope.

### Semi-quantitative RT-PCR analysis

Semi-quantitative RT-PCR was performed to determine the expression level of different proinflammatory molecules. Experiments were performed by using a RT-PCR kit from BD Clontech as described earlier^44^,^47^,^48^. In brief, the cells from stimulated and unstimulated sets of experiments were harvested, RNA was isolated and purified by using the Qiagen mini kit followed by digestion with DNase. cDNA was prepared from 1 μg of purified RNA by reverse transcribed using oligo(dT)_12–18_ as primer and MMLV reverse transcriptase (BD Clontech) in a 20 μl reaction mixture. The resulting cDNA was diluted and amplified using Titanium TaqDNA polymerase using the following primers. The following primers were used to amplify mouse proinflammatory molecules: iNOS: sense: 5′-CCC TTC CGA AGT TTC TGG CAG CAG C-3′, antisense: 5′-GGC TGT CAG AGC CTC GTG GCT TTG G-3′; IL-1B: sense: 5′- ATG GCA ACT GTT CCT GAA CTC AAC T -3′; antisense: 5′- CAG GAC AGG TAT AGA TTC TTT CCT TT-3′; IL-6: sense: 5^′^-TGG AGT CAC AGA AGG AGT GGC TAA G-3′; antisense: 5′-TCT GAC CAC AGT GAG GAA TGT CCA C-3′; TNF-α: sense: 5^′^-TTC TGT CTA CTG AAC TTC GGG GTG ATC GGT CC-3′; antisense: 5^′^-GTATGA GAT AGC AAA TCG GCT GAC GGT GTG GG-3′; GAPDH: sense: 5′-GGT GAA GGT CGG TGT GAA CG-3′, antisense: 5′-TTG GCT CCA CCC TTC AAG TG-3′. Amplified products were analyzed with 1.8% agarose gels electrophoresis and visualized with UV Transilluminator. GAPDH was used to ascertain that an equivalent amount of cDNA was synthesized from different samples. The relative expression of iNOS, TNF-α, IL-6 and IL-1β (IL-1β/GAPDH) was measured after scanning the bands with a Fluor Chem 8800 Imaging System (Alpha Innotech).

### Assay for NO Synthesis

Synthesized NO was determined by assay of culture supernatants for nitrite, a stable reaction product of NO with molecular oxygen, using Griess reagent^49^,^50^.

### Immunoblotting assays

The harvested cells were lysed in RIPA buffer [PBS (1X), 1% nonidet-40, 0·5% sodium deoxycholate, 0·1% sodium dodecyl sulphate, 0·5% protease inhibitor cocktail (Sigma)] in ice. Protein content was estimated using Protein assay dye reagent concentrate (Bio-Rad) following manufacturer’s protocol. Immunoblotting was performed and detected by fluorescence detection in the Odyssey infrared imaging system (LI-COR Biosciences, Lincoln, NE) as described previously^51^.

### Assay of NF-κB, IL-1β, TNF-α and IL-6 promoter-driven reporter activity

Cells plated at 50–60% confluence in 12-well plates were cotransfected with 0.25 μg of NF-κB, pIL-1β-Luc, pIL-6-Luc, pTNF-α-Luc and 25 ng of pRL-TK using Lipofectamine Plus (Invitrogen). After 24 h of transfection, cells were treated with different concentrations of lignin, lig-g-POZ and 3-AT-c-lig-g-POZ for 2 h prior to the addition of LPS under serum free condition for 6 h. The luciferase activities were estimated in cell extracts using the Dual Luciferase kit (Promega) in a TD-20/20 Luminometer (Turner Designs)^47^,^49^,^52^. Relative luciferase activity was represented as the ratio of firefly luciferase value:Renilla luciferase value × 10^−3^.

### Electrophoretic mobility shift assay

Electrophoretic mobility shift assay (EMSA) was performed using nuclear extracts^45^,^53^,^54^ with some modifications. IRDye infrared dye end-labeled oligonucleotides (5′-AGT TGA GGG GAC TTT CCC AGG C-3′) for NF-κB was procured from Licor Biosciences. Six micrograms of nuclear extract was incubated with infrared-labeled probe in binding buffer for 20 min. Further, samples were separated on a 6% polyacrylamide gel electrophoresis with 0.25 × TBE buffer (Tris borate-EDTA). Images were captured and analyzed with Odyssey Infrared Imaging System (LI-COR Biosciences).

### Cell Viability Measurement

Mitochondrial activity was measured with the 3-(4,5-dimethylthiazol-2-yl)-2,5-diphenyltetrazolium bromide (MTT) assay (Sigma). In brief, cells (5 × 10^5^ cells/ml) were seeded on 24-well culture plates with 500 μl medium and treated with lignin, lig-*g*-POZ, 3-AT-c-lig*-g*-POZ and final formulated nanocomposite product in each separate sets of experiments. After various treatments, 300 μl of culture medium were removed from each well, and cells were incubated with 20 μl of MTT solution (0.5 mg/ml) for 1 h. The supernatant was removed and the formation of formazon crystals was measured at 540 nm with a microplate reader.

### Lactate Dehydrogenase Assay

Lactate dehydrogenase Activity (LDH) was measured in the culture medium after 24 h using the direct spectrophotometric assay using an assay kit from Sigma.

### Antimicrobial Assay

The antimicrobial activity of lignin, lig-*g*-POZ and 3-AT-c-lig-*g*-POZ copolymer were assessed against bacterial strains as *E. coli, P. aeruginosa, S. typhi, K. pneumoniae, S. aureus, S. epidermidis* and fungal strains *C. albicans* and *C. tropicalis*. The minimum inhibitory concentration (MIC) of all synthesized copolymers against the pathogens were assessed using micro dilution plate method followed by National Committee for Clinical Laboratory Standards Institute (CLSI). A 200 μL of total volume in each well was made by amending the growth medium (RPMI 1640 for fungal strains and Muller Hinton broth for bacterial strains) with different concentrations of individual copolymers (512–1.0 μg/mL) using appropriate inoculum dose (3.5 × 10^6^ CFU/mL). Two wells of the plate used as growth (without copolymer) and sterility (without inoculum) controls. The plates were incubated at 30 °C with 18 h for fungal growth and 37 °C with 18 h for bacterial growth. All experiments were repeated in triplicates.

Simultaneously, the antibiofilm activity was checked against *Pseudomonas aeruginosa*, a most opportunistic wound forming pathogen. Biofilm eradication ability was determined in the presence of copolymers individually with 3AT-c-lig-*g*-POZ and 3AT-c-lig-*g*-POZ with antibiotics (piperacillin/tazobactum). Cells were grown in a 30 mm polysterene plate containing 2 mL MHB (Muller Hinton Broth) medium with inoculum dose 3.5 × 10^6^ CFU/mL, incubated for 48 h at 37 °C and a glass cover slip was dropped into the each plate with treatment and untreated plate was used as control. The formation of biofilm on glass cover slip was determined by crystal violet assay and images were captured in Atomic Force Microcopy (AFM) after gently washed with PBS (1X).

### *In-vivo* assay

All experiments were performed after deriving approval from the ethical committee constituted by the North Bengal University. The necessary guidelines and regulations of the said committee were followed while conducting the experiments. In brief, studies were done with Sprague Dawley rats, maintaining the sex ratio of 1: 1, and one animal per cage (Tarson, India) to prevent aggression, if any, of one towards the other of the same sex or opposite. The cages bedded with rice husk were kept in animal enclosure under proper conditions of temperature (25 + /−2 °C), humidity (55 + /−5%), and photoperiod (12 h). Food pellets (Pranav Agro Pvt. Ltd., India) and filtered tap water (Aquaguard Eureka Forbes, India) were fed to the animals *ad-libitum*.

### Burning procedure

The method of burning was similar to the techniques reported by earlier authors^55^,^56^ with slight modification to a little extent. First of the region of the bodies of animals, where burning is intended, the fur/hairs were shaved using sterile scissor and razor. The animals were anaesthetized with ether-chloroform (1:3). A fire protective cloth having a window of 1 inch^2^ was held tightly over the shaved region on the back of the anaesthetized animal followed by spreading of 0.2 ml of 95% ethanol in the window space. The spread ethanol was lit up with a matchstick and left to burn for 15 s. Instantaneously, after the burn episode, 0.5 ml of physiological saline Solution (PSS; 0.85% NaCl) was injected intraperitoneally to enable recovery from the burn shock.

### External Infection in the burn-wound site

Infection of the animals (that have undergone burn) was done by inoculating the burn area with the bacterial strain, *Pseudomonas aeruginosa* HW01. The strain was isolated from the hospital waste water following methods described earlier^57^. Overnight grown HW01 cells in Luria broth (Himedia, India) were centrifuged to obtain the cell pellet. The cell pellet was suspended in sterile PBS, centrifuged followed by re-suspension in PBS. After another round of washing in PBS, cell pellet was finally diluted in the same buffer to obtain a cell density of 10^9^ ml^−1^. A measured volume of 200 μl cell suspension was used to infect single burn zone in each animal. Sterile cotton gauze (single layer) was detained over the burn-area to aid effective absorption of the inoculum for a period of 10 min before any other application.

### Topical treatment of burn-afflicted site

After completion of the external infection and removal of the gauze, applications of materials for treatment were done. There were five groups [six animals (1:1 sex ratio) per group] corresponding to five different treatments of the infected burn-wounds. Gr-I was treated with lignin nanocomposite alone. Gr-II was treated with lignin nanocomposite mixed with dry piperacillin/tazobactum (Lupin ltd., India). Gr-III was treated with piperacillin/tazobactum alone. Gr-IV was treated with silver nitrate and chlorohexidine gluconate cream. Gr-V was treated with a spray of PSS alone. Separate sterile applicator sticks were used in different treatments. All the sixty rats, distributed equally in five different groups, that had undergone specific treatment were examined every day (till the near complete healing of burn wounds in any one of the treated groups) for measuring the percent residual wound area to obtain quantitative data of wound healing.

### Qualitative determination of the pathogen load in the burn-wound site

Microbiological evaluation was carried out using “swabs” in the injury area from the 2nd day of the burning experiment. The sample was collected with the cotton swabs, then the cotton swabs were dipped in 1 mL PBS containing solution. After that the swab drained PBS was asceptically spreaded onto King’s Medium A Base (HIMEDIA, M1543-500G) plate. After 24 h of incubation, spread-plates in triplicate were evaluated for *Pseudomonas aeruginosa* HW01 colonies. This routine check was continued till the healing/non appearance of *Pseudomonas aeruginosa* HW01 to evaluate the degree of contamination of injuries.

### Weight and food intake analysis of the burn-inflicted Sprague Dawley rats

The weights of the rats were examined daily and the food intake by rats was also noted regularly. All the data were collected till the recovery of the treated burn wounds.

### C-reactive protein (CRP) level determination following the infliction of burn and infection

The blood of the rats were collected from the tail vein in the sterile 0.25 mL PCR tubes. Then the collected blood was placed in vertical position for settling down of the blood corpuscles for a period of 30-45 min. The blood samples were then centrifuged for 5 min at 1500 g in order to separate the serum. The separated serum samples were collected in sterile PCR tubes. The collected serum was used to determine qualitative and Semi-quantitative CRP level using RHELAX-CRP latex reagent (Tulip Diagnostics (P) Ltd., India). PSS saline was prepared for diluting the specimen sample. For quantitative CRP measurement 1:2, 1:4, 1:8, 1:16, 1:32 and so on dilutions were made and RHELAX-CRP latex reagent was mixed with diluted samples. Then the mixture was mixed properly by the mixing stick and the agglutination reaction was observed macroscopically within 2-3 min.

### Antimicrobial susceptibility test

Antibiotic -resistance/susceptibility profile of P. aeruginosa HW01 was determined following the recommendation of CLSI^58^. Disc-diffusion method employing Kirby-Bauer technique was applied on Mueller-Hinton agar plates. Muellar-Hiton agar (MHA) medium prepared as per manufacturer’s instruction (HiMedia, India) was dispensed at 20 mL per plate. Log-phase culture of HW01 (equivalent to 0.5 McFarland standard) was spread-plated onto MHA plates. Antibiotic disks (HiMedia, India), amikacin-AK (30 μg), aztreonam- AT (30 μg), cefepime-CPM (30 μg), ceftazidime-CAZ (10 μg), ciprofloxacin-CIP (5 μg), colistin-CL (10 μg), doripenem-DOR (10 μg), levofloxacin-LE (5 μg), meropenam-MRP (10 μg), piperacillin/tazobactum-PIT (100/10 μg), ticarcillin-TI (75 μg), and tobramicin-TOB (10 μg) were suitably placed on the spread plates and incubated for 24 h at 37 0 C. Resistance (R)/Sensitive (S) to a particular antibiotic was determined after measuring the inhibition zone and inferred following comparison with the zone size given in the interpretive chart of EUCAST/NCCLS.

### Statistical analysis

The percent residual wound areas (PRWA) for each treatment group (n = 6) on different day (0/2nd/4th/6th/8th/10th/12th/14th day following treatment) were averaged and recorded as mean ± SD. In order to analyze the differences in PRWA between five different treatment groups on 14th day, One-way ANOVA with post-hoc Turkey HSD, Scheffe, Bonferroni and Holm multiple comparison tests were done using Test calculator (astatsa.com/OneWay_Anova_with_TurkeyHSD/). From post-hoc Turkey results, the p-value corresponding to the F-statistic of one-way lower than 0.01 were accepted as pairs of treatment being significantly different. From Bonferroni and Holm results, only pairs relative to treatment A (Control) were simultaneously compared [A (Gr-V, treated with a spray of PSS alone) Vs B (Gr-II, treated with lignin nanocomposite mixed with dry piperacillin/tazobactum); A Vs C (Gr-I, treated with lignin nanocomposite alone); A Vs D (Gr-IV, treated with silver nitrate and chlorohexidine gluconate cream); A Vs E (Gr-III, treated with piperacillin/tazobactum alone); B vs C; B vs D, and B vs D] to draw Bonferroni and Holm inferences (insignificant or significant).

## Additional Information

**How to cite this article:** Mahata, D. *et al*. Lignin-*graft*-Polyoxazoline Conjugated Triazole a Novel Anti-Infective Ointment to Control Persistent Inflammation. *Sci. Rep.* **7**, 46412; doi: 10.1038/srep46412 (2017).

**Publisher's note:** Springer Nature remains neutral with regard to jurisdictional claims in published maps and institutional affiliations.

## Supplementary Material

Supplementary Files
