# Supplementary material for: Lignin-graft-Polyoxazoline Conjugated Triazole a Novel Anti-Infective Ointment to Control Persistent Inflammation
Source: Sci Rep. 2017 Apr 12;7:46412. doi: 10.1038/srep46412 (PMC5401907; doi:10.1038/srep46412)
Supplement: Supplementary Files [file srep46412-s1.pdf]

Supporting Information

Lignin-*graft*-Polyoxazoline Conjugated Triazole a Novel Anti-Infective Ointment to Control Persistent Inflammation

Denial Mahata,[a] Malabendu Jana,[b] Arundhuti Jana,[b] Abhishek Mukherjee,[c] Nibendu Mondal,[d] Tilak Saha,[d] Subhajit Sen,[d] Golok B. Nando,[a] Chinmay K. Mukhopadhyay,[c] Ranadhir Chakraborty,[d] Santi M. Mandal[a,*]

[a]Central Research Facility, Rubber Technology Centre, Indian Institute of Technology Kharagpur, Kharagpur 721302, WB, India, [b]Department of Neurological Sciences, Rush University Medical Center, Chicago, IL, USA. [c]Special Centre for Molecular Medicine, Jawaharlal Nehru University, New Delhi 110 067, India. [d]OMICS Laboratory, Department of Biotechnology, University of North Bengal, Siliguri- 734 013, WB, INDIA.

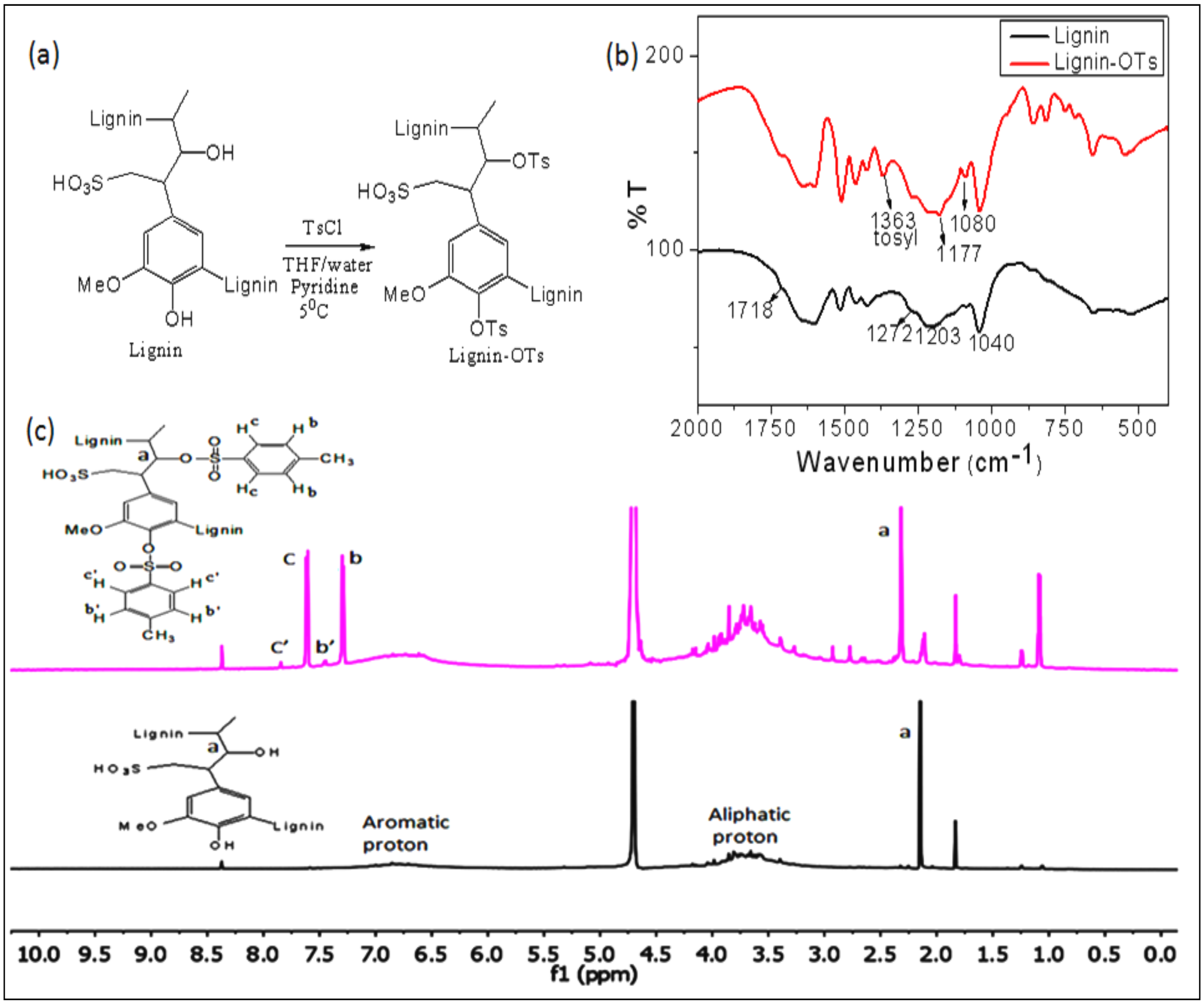

Figure S1. Synthesis and characterization of tosylated lignin macroinitiator. Synthesis procedure of tosylated lignin (a), FTIR spectrum (b) and ^{1}HNMR spectrum of lignin-OTs macroinitiator.

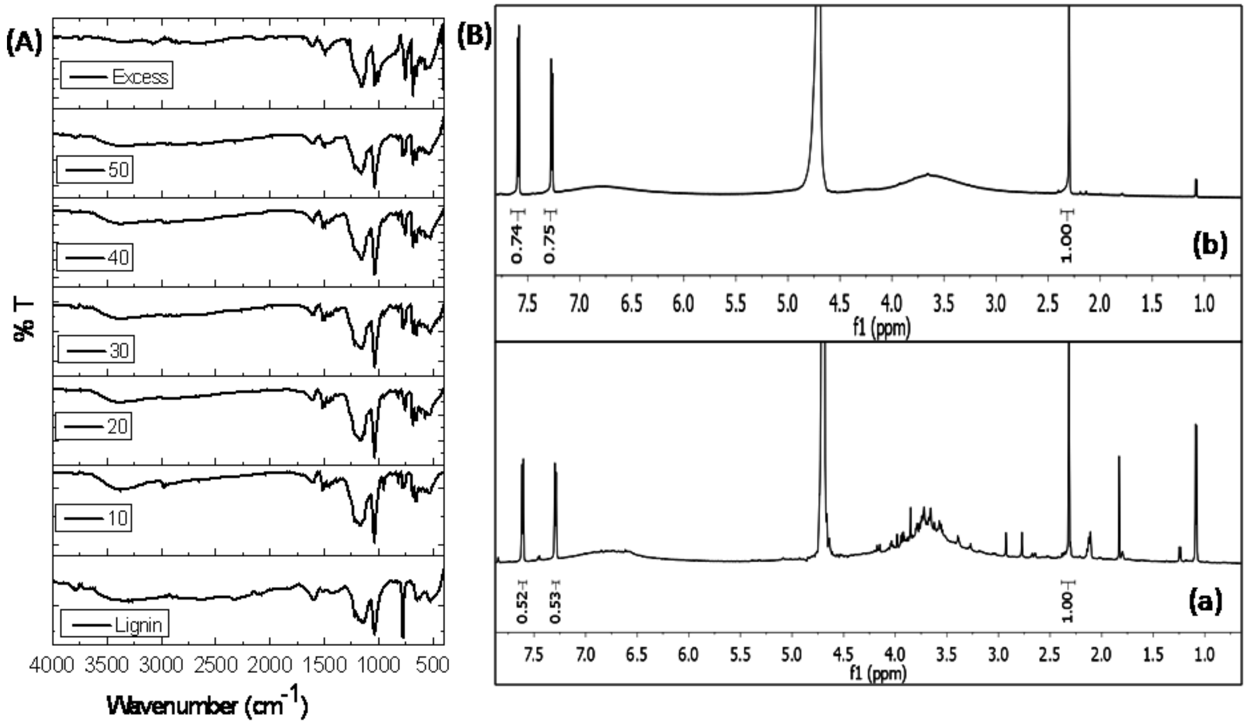

Figure S2. Determination of degree of tosylation in lignin-OTs macroinitiator. FTIR spectrum (A) of different weight (%) of feeding tosyl chloride in reaction. The ^{1}HNMR spectrum (B) of lignin-OTs partially substituted (a) and fully substituted (b) macroinitiator.

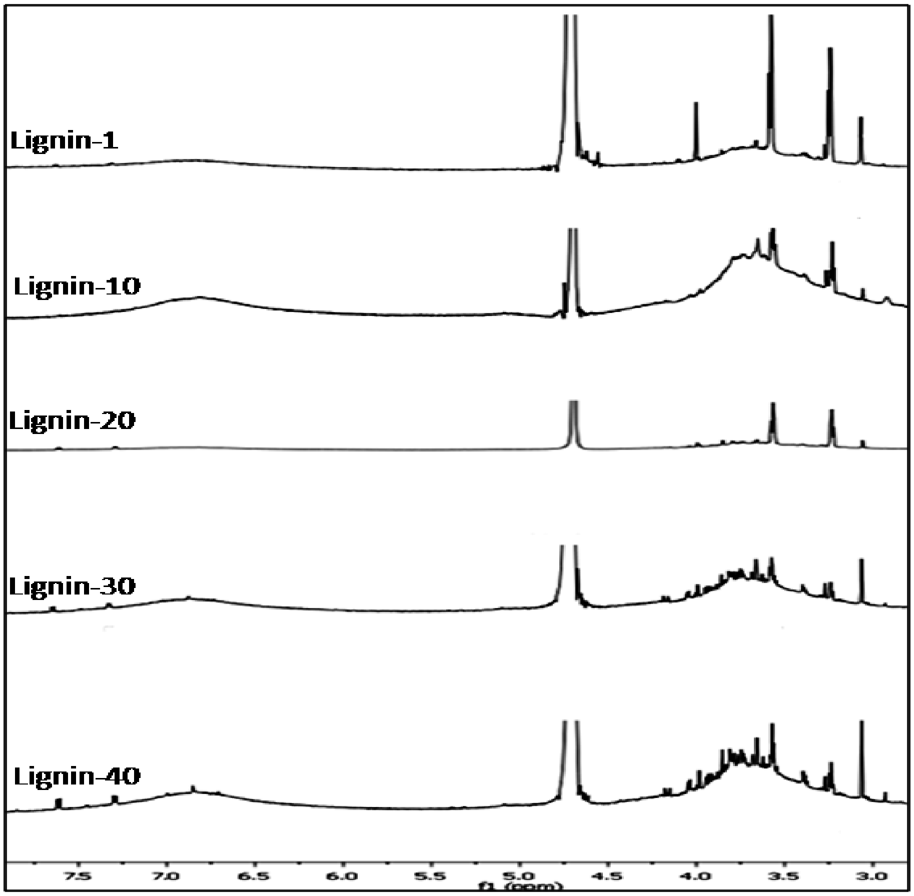

Figure S3. ^{1}H NMR spectrum of lignin-*g*-POZ copolymers with different weight (%) lignin content in D_2O.

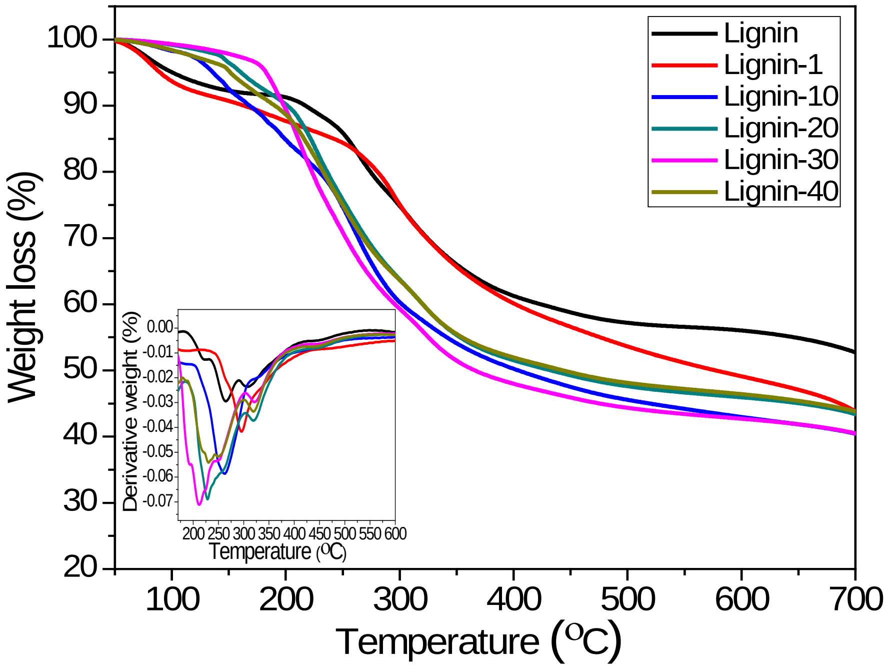

Figure S4. Thermo-gravimetric analysis of lignin-*g*-POZ copolymer. TGA and DTGA (inset) curve (a) of different weight (%) lignin in copolymer.

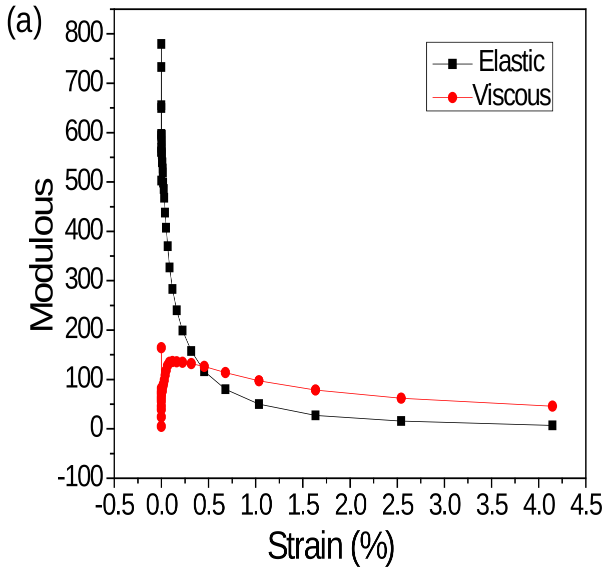

Figure S5. Strain vs modulous (a) and time vs modulus plot (b) of optimized 20 wt% lignin in Lignin-*g*-POZ in copolymer hydrogel.

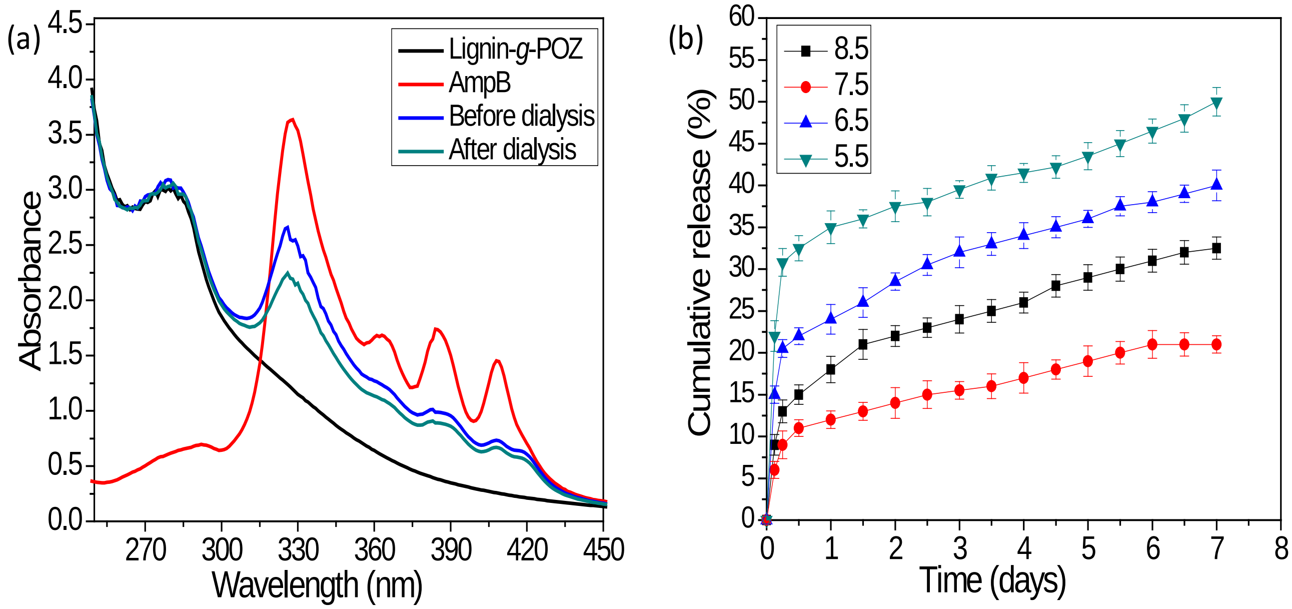

Figure S6. Drug loading and release profile. UV-visible absorption spectrum of AmpB loaded lignin-*g*-POZ copolymer hydrogel (a). *In vitro* release of amphotericin –B (AmpB) from hydrogel with different pH at 8.5, 7.5, 6.5 and 5.5 in buffer solution.

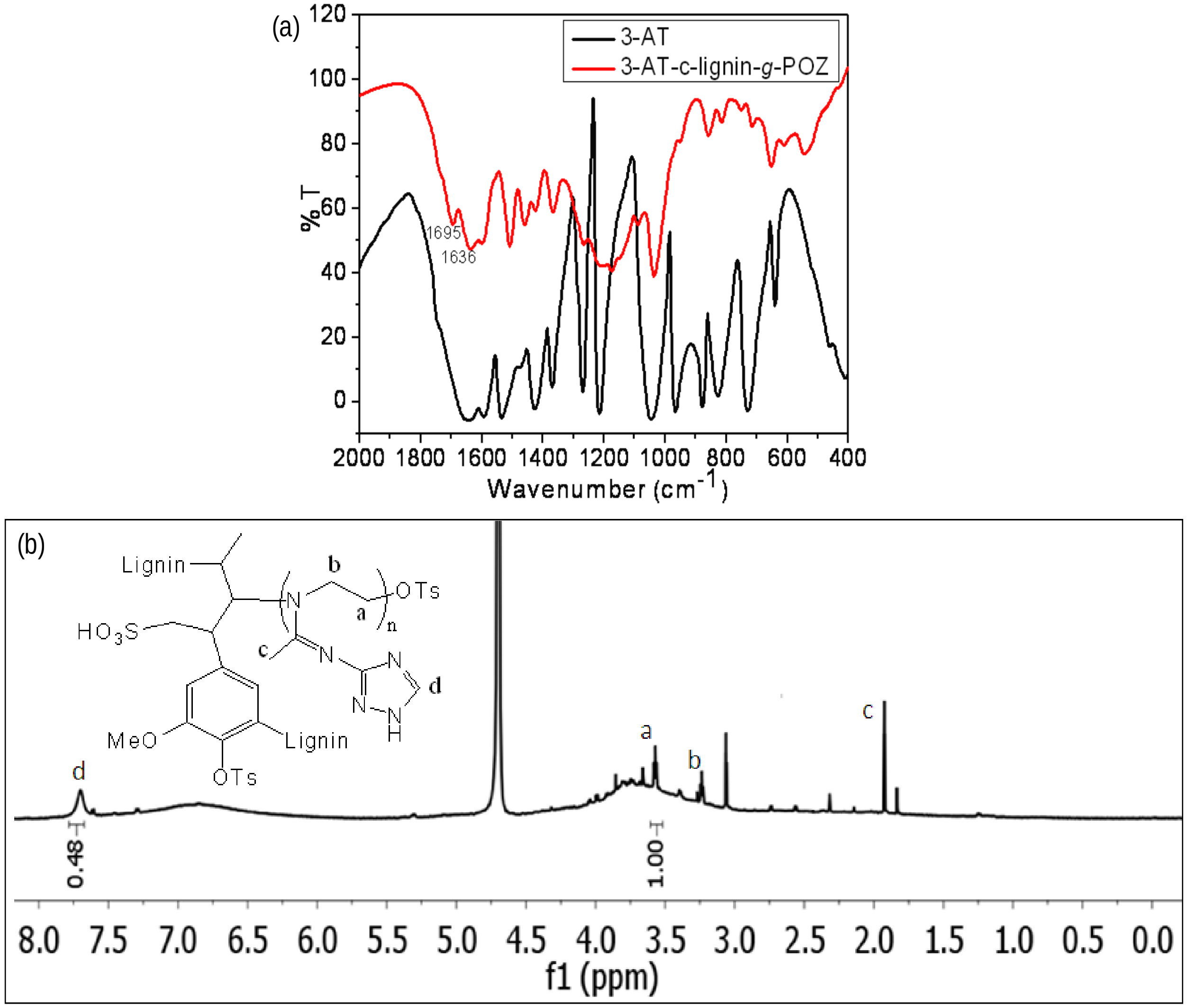

Figure S7. Characterization of 3-AT-c-lignin-*g*-POZ copolymer. FTIR spectrum of 3-AT and 3-AT-c-lig-*g*-POZ (a) and ^{1}HNMR spectrum of 3-AT-c-lig-*g*-POZ (b).

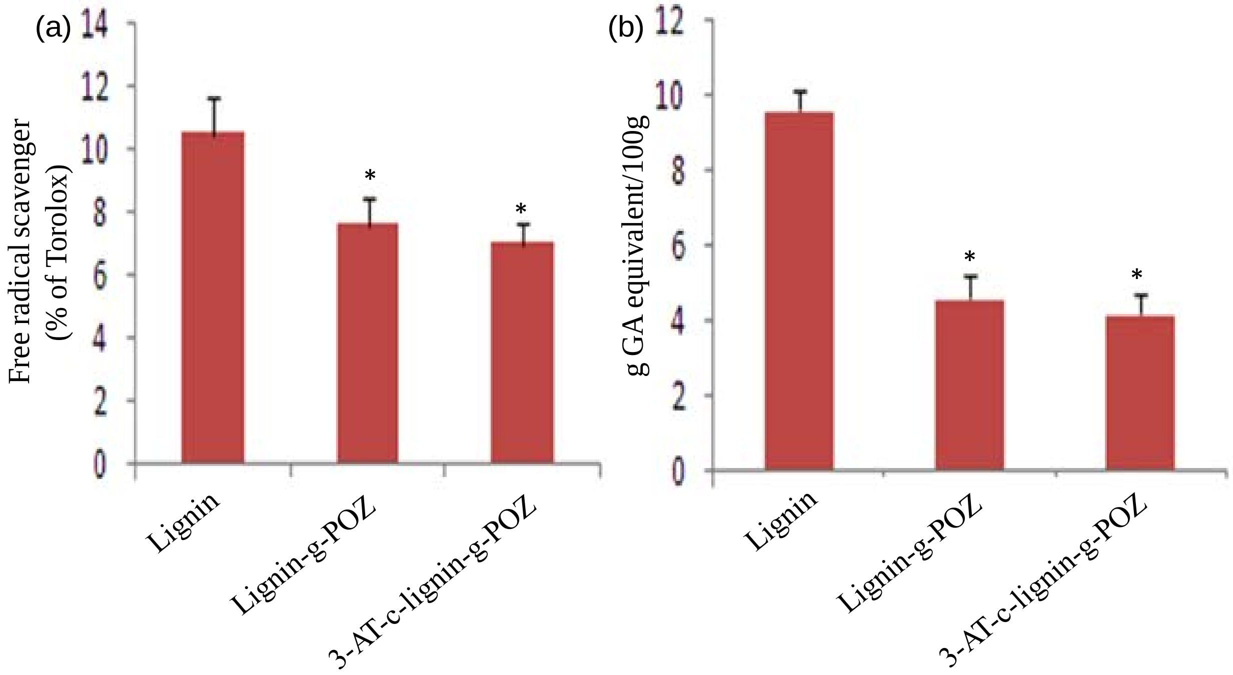

Figure S8. Free radical scavenging activity (a) and total phenol content (b) of lignin, lig-*g*-POZ and 3-AT-c-lig-*g*-POZ copolymer. Data are the mean of triplicates (n=3) and bars, represent standard deviation (±SD). *The level of significance ($p < 0.05$) was determined with respect to lignin.

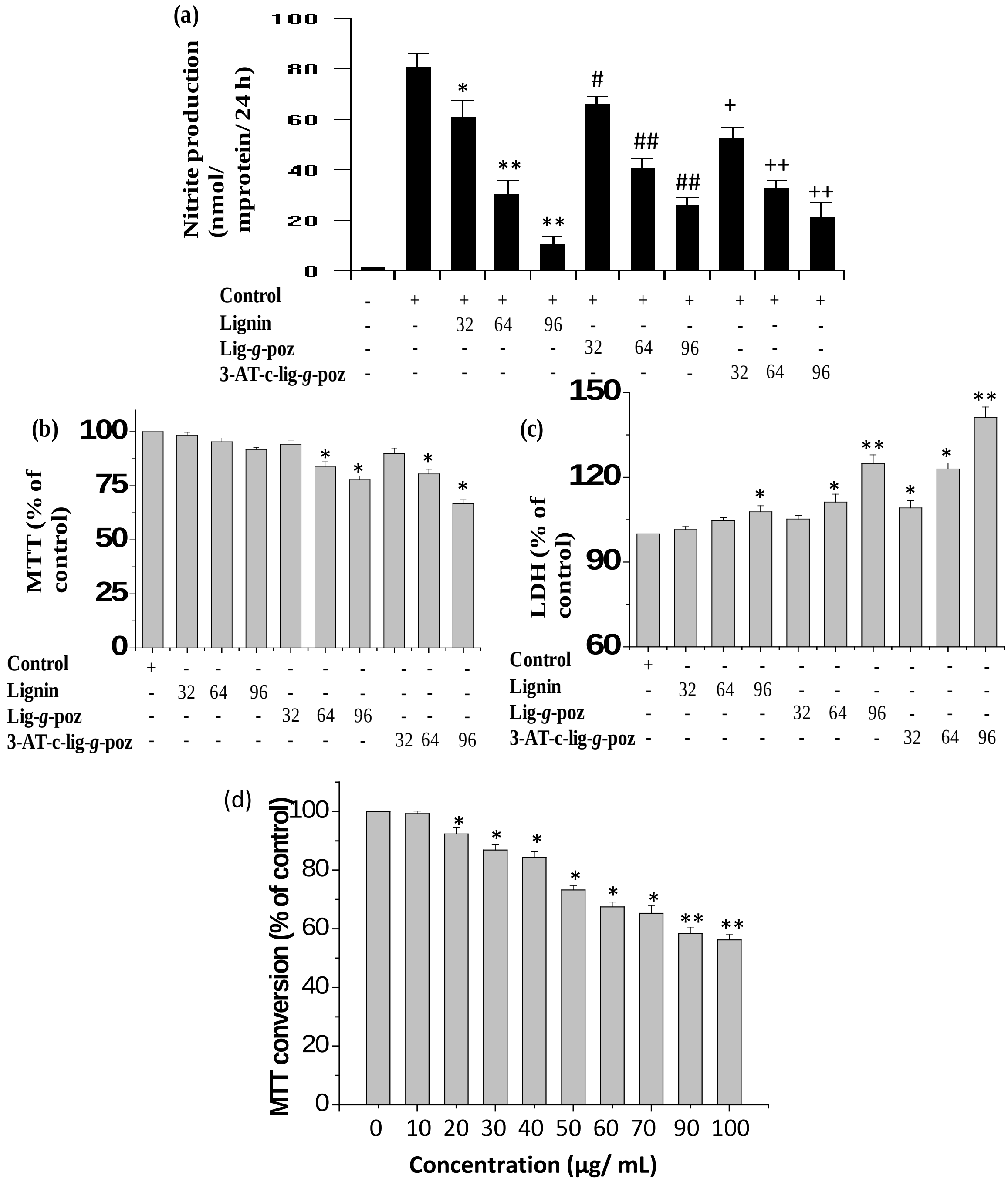

Figure S9. Lignin derivatives inhibited the LPS-induced-NO production in macrophages: RAW cells were preincubated with different concentrations of lignin, lignin-*g*-POZ and 3-AT-c-lignin-*g*-POZ for 2 h under serum free conditions were stimulated with 1 µg/mL of LPS and after 24 h, the nitrite concentrations were measured (A). Results are mean ± S.D. of three different experiments. *p< 0.05 and **p<0.01 *vs* cells treated with LPS, #p < 0.05 and ##p<0.01 *vs* cells treated with LPS, +p < 0.05 and ++p<0.01 *vs* cells treated with LPS in the absence of lignin

derivatives. After 12 h of stimulation, cell viability was examined by the metabolism of MTT (B) and the release of LDH (C). Values obtained from the control group served as 100%. Data obtained in other groups calculated as percent of control accordingly and the level of significance (*$p < 0.05$, **$p < 0.01$) determined compared to control. Dose dependent cytotoxicity analysis using MTT assay. Cell viability was monitored in a range of final formulated nanocomposite dose (10-90 µg/ mL) against human embryonic kidney (HEK 293) cell line (D). Results are mean ± S.D. of three different experiments and the level of significance (*$p < 0.05$, **$p < 0.01$) was determined with respect to control.

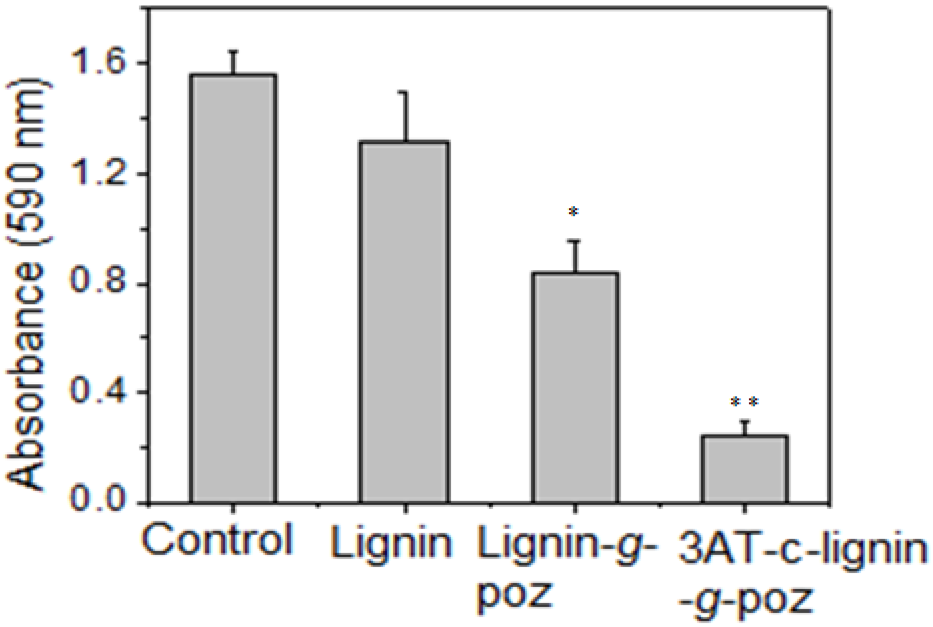

Figure S10. Biofilm quantification with CV stain of lignin, lig-*g*-POZ and 3AT-c- lig-*g*-POZ. Data are the mean of triplicates and scale bars represent standard deviation (±SD). The level of significance (*$p < 0.05$, **$p < 0.01$) was determined with respect to control.

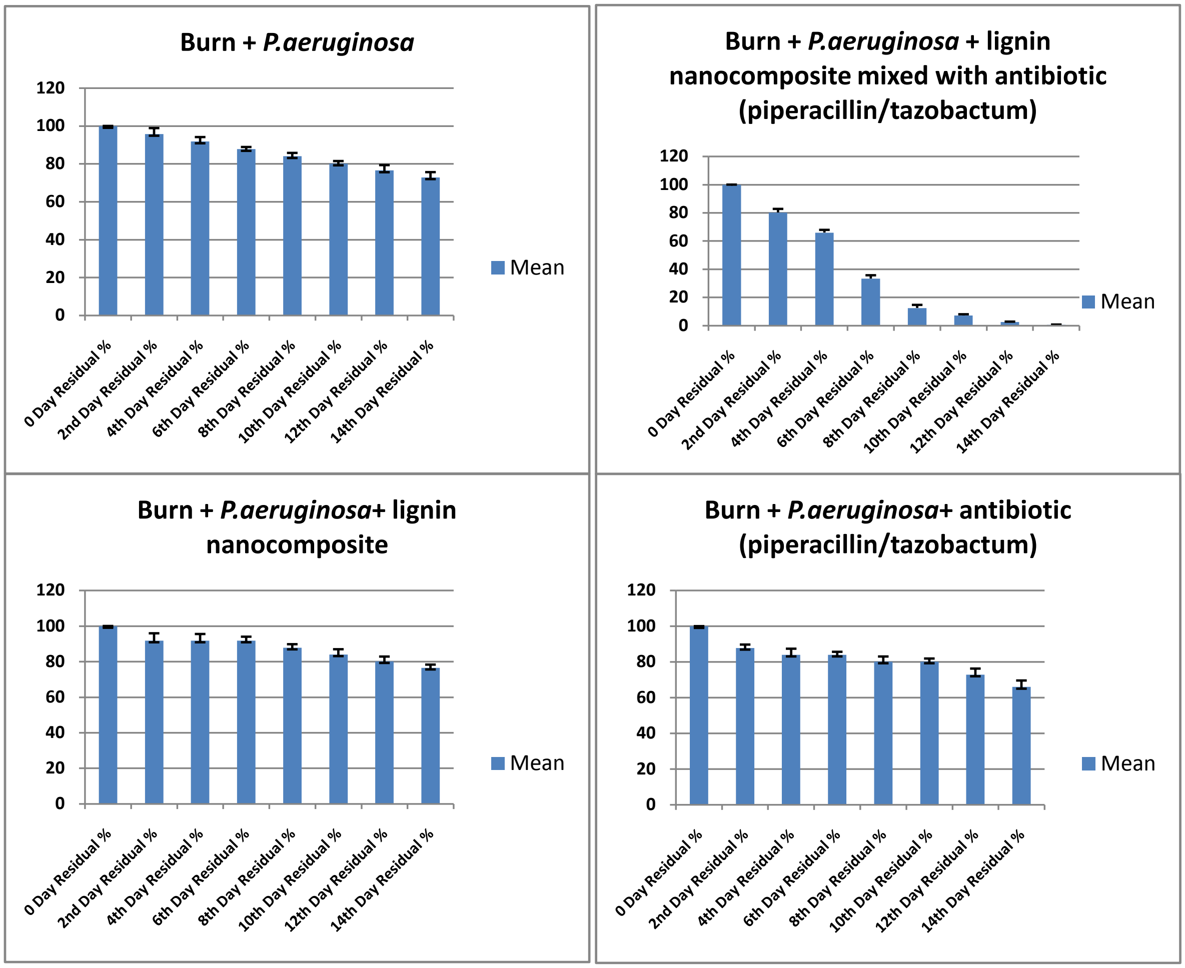

Fig. S11 Wound healing in terms of persistence of wound area (%). Treatment of infected burn wounds were grouped in four categories: (a) no treatment; (b) treated with lignin nanocomposite + antibiotic (1 mL mixture contained 64 µg lignin nanocomposite and 56/7 µg piperacillin/tazobactam); (c) treated with only lignin nanocomposite (64 µg/ mL); (d) treated with antibiotics (1 mL contained 56/7 µg piperacillin/tazobactam).

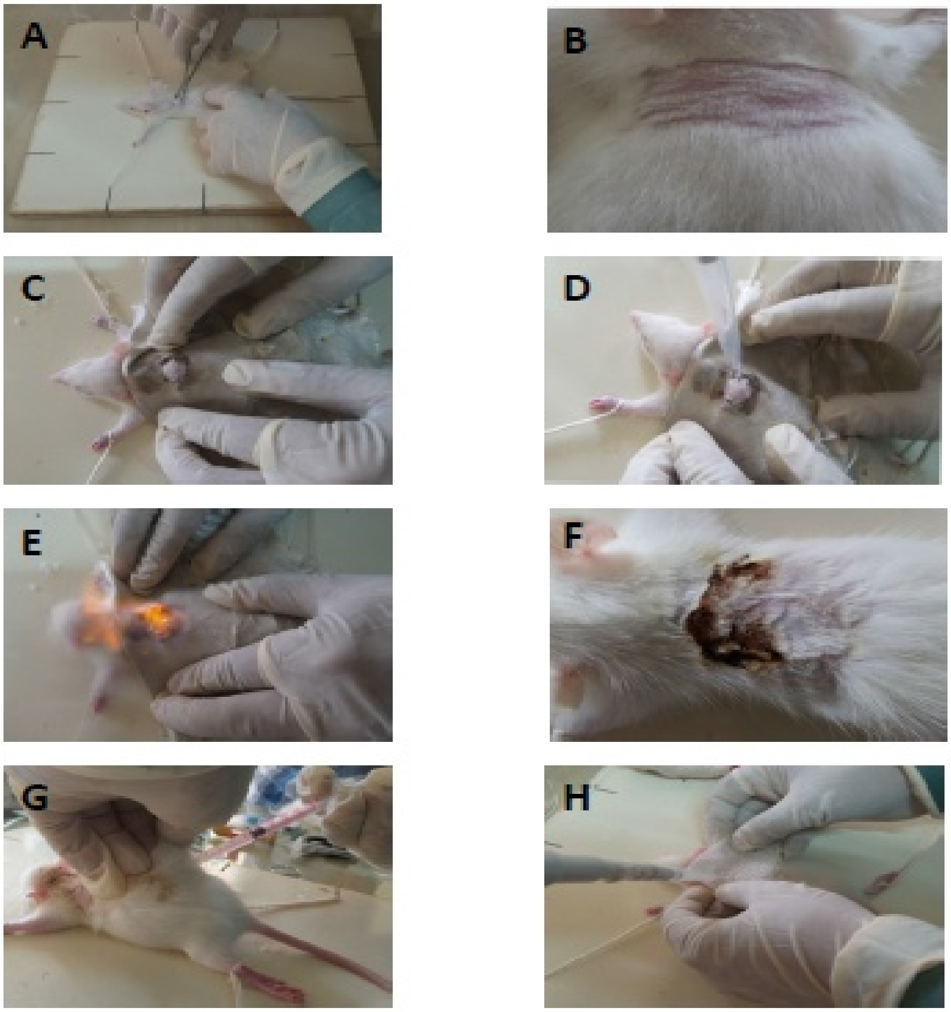

Figure S12. Process of burning and post burning infection with MDR *Pseudomonas aeruginosa* HW01 in Spague Dawley Rat. (A)- Shaving of the back region to burn; (B)- Shaved area; (C)- covering of the outer regions of prospective burn with thermal insulator cloth; (D)- spreading of 95% ethanol; (E)- burning with flame for 15 seconds; (F)- post burn image; (G)- injecting 500ul normal saline intraperitoneally; (H)- infecting the burn spot with *P. aeruginosa* HW01 culture (2 x 10^8 c.f.u).

Table S1. Number average molecular weight (M_n) of each polyoxazoline chain length and their thermal properties of Lig-*g*-POZ copolymer with different weight (%) lignin content.

Sample Name	M_n of each POZ chain length (Da) by ^{1}HNMR	Maximum degradation temperature (°C)	Glass transition temperature (T_g)	Melting Temperature (°C)	Heat of Enthalpy (ΔH) (J/gm)
Lignin-1	5644	295	102	128	-46.1
Lignin-10	694	261	129	147	-83.2
Lignin-20	593	227	_	148	-107.4
Lignin-30	407	212	_	148	-236.5
Lignin-40	282	231	_	148	-380.1
Lignin-100	00	264	_	142	-175.2

Table S2. Minimum inhibitory concentration of lignin copolymers against bacteria and fungi. Data are represented as µg/mL, "nd' not detected up to 1 mg/mL.

Compounds	*E. coli* ATCC25922	*P. aeruginosa* ATCC27863	*S. typhi* MTCC734	*K. penumonia* ATCC714	*S. aureus* ATCC21737	*S. epidermidis* NCIM 2493	*C. albicans* NCIM 3557	*C. tropicalis* NCIM 3110
Lignin	nd	nd	nd	nd	nd	nd	nd	nd
Lig-*g*-Poz	1000	nd	nd	nd	nd	nd	nd	nd
3-AT-c-lig-*g*-Poz	250	500	500	1000	500	500	250	250
Piperacillin/tazo-bactum	1	2	4	16	0.5	0.5	nd	nd
Gentamicin	1	4	2	0.5	8	4	nd	nd

Table: S3. Microbiological evaluation of presence of *P. aeruginosa* in the post-burn wound and determination of C-reactive protein (CRP) level in the blood of the experimental rats.

A. Evaluation of presence of *P. aeruginosa* in different days of post-burn wounds in rats. +++, very high presence [$10^4 – 10^5$ c.f.u./ml of swab drained PBS (SDP)]; ++, high presence [$10^3 – 10^4$ c.f.u./ml SDP]; +, moderate presence $10^2 – 10^3$ c.f.u./ml SDP]; +/- ,negligible presence **(**$10 – 10^2$ c.f.u./ml SDP]; -, no presence detected [0-10 c.f.u./ml SDP].

Treatment	01-day	02-day	03-day	04-day	05-day	06-day	07-day
Burn + *P.aeruginosa* + lignin nanocomposite mixed with antibiotic (piperacillin/tazobactum) [1 mL mixture contained 64 µg lignin nanocomposite and 56/7 µg piperacillin/tazobactam]	++	++	+/-	-	-	-	-
Burn+ *P.aeruginosa*	+++	+++	+++	++	+	+/-	+/-
Burn+ *P.aeruginosa* + lignin nanocomposite (64 µg/ mL)	+++	+++	++	++	++	+/-	+/-
Burn+ *P.aeruginosa* + antibiotic (piperacillin/tazobactum) [1 mL contained 56/7 µg piperacillin/tazobactam]	++	++	++	++	++	+	+/-

B. Level of C-reactive protein in the blood of animals (both treated and untreated) on second and fourth day following burn and infection with *P. aeruginosa.*

Treatment	Agglutinations in different dilutions of the serum					
	1	1:2	1:4	1:8	1:16	mg/ml
**02- day**						
Burn + *P.aeruginosa* + lignin nanocomposite mixed with antibiotic (piperacillin/tazobactum) [1 mL mixture contained 64 µg lignin nanocomposite and 56/7 µg piperacillin/tazobactam]	+	+	+	+	-	**4.8**
Burn+ *P.aeruginosa*	+	+	+	+	-	**4.8**

Burn+ *P.aeruginosa* + lignin nanocomposite (64 µg/ mL)	+	+	+	+	+	**9.6**
Burn+ *P.aeruginosa* + antibiotic (piperacillin/tazobactum) [1 mL contained 56/7 µg piperacillin/tazobactam]	+	+	+	+	+	**9.6**
**04- day**						
Burn + *P.aeruginosa* + lignin nanocomposite mixed with antibiotic (piperacillin/tazobactum [1 mL mixture contained 64 µg lignin nanocomposite and 56/7 µg piperacillin/tazobactam]	**-**	**-**	**-**	**-**	**-**	**0**
Burn+ *P.aeruginosa*	+	+	+	+	+	**9.6**
Burn+ *P.aeruginosa* + lignin nanocomposite (64 µg/ mL)	+	+	+	+	+	**9.6**
Burn+ *P.aeruginosa* + antibiotic (piperacillin/tazobactum) [1 mL contained 56/7 µg piperacillin/tazobactam]	+	+	+	+	+	**9.6**
